# Objective Definition of Rosette Shape Variation Using a Combined Computer Vision and Data Mining Approach

**DOI:** 10.1371/journal.pone.0096889

**Published:** 2014-05-07

**Authors:** Anyela Camargo, Dimitra Papadopoulou, Zoi Spyropoulou, Konstantinos Vlachonasios, John H. Doonan, Alan P. Gay

**Affiliations:** 1 Institute of Biological, Environmental and Rural Sciences, Aberystwyth University, Gogerddan, Aberystwyth, Ceredigion, United Kingdom; 2 Aristotle University of Thessaloniki, Faculty of Science, School of Biology, Department of Botany, Thessaloniki, Greece; Universidad Miguel Hernández de Elche, Spain

## Abstract

Computer-vision based measurements of phenotypic variation have implications for crop improvement and food security because they are intrinsically objective. It should be possible therefore to use such approaches to select robust genotypes. However, plants are morphologically complex and identification of meaningful traits from automatically acquired image data is not straightforward. Bespoke algorithms can be designed to capture and/or quantitate specific features but this approach is inflexible and is not generally applicable to a wide range of traits. In this paper, we have used industry-standard computer vision techniques to extract a wide range of features from images of genetically diverse *Arabidopsis* rosettes growing under non-stimulated conditions, and then used statistical analysis to identify those features that provide good discrimination between ecotypes. This analysis indicates that almost all the observed shape variation can be described by 5 principal components. We describe an easily implemented pipeline including image segmentation, feature extraction and statistical analysis. This pipeline provides a cost-effective and inherently scalable method to parameterise and analyse variation in rosette shape. The acquisition of images does not require any specialised equipment and the computer routines for image processing and data analysis have been implemented using open source software. Source code for data analysis is written using the R package. The equations to calculate image descriptors have been also provided.

## Introduction

The goal of this study was to use a computer vision and data mining approach to compare the rosette shapes of the founders of a Multiparent Advanced Generation Inter-Cross (MAGIC) population. A genetic analysis of the same population was performed and reported previously [1]. Objective computer-aided phenotyping has been proposed as a solution to the genotype-phenotype bottleneck [2], but there remain numerous technical challenges with regard to its implementation at the whole organism level. However, there has been little exploration of the ability of computer vision techniques to define and discriminate between phenotypes.

We chose *Arabidopsis* rosettes as our experimental material for three main reasons. First, the rosettes in this species (under our growth conditions) grow close to the ground and can be treated essentially as 2-D objects, simplifying image acquisition and processing. Second, previous studies indicate that there is significant shape variation between accessions. Natural variation in continuously varying traits has been shown for morphological traits and for responses to stimuli. Examples of the former are morphological comparisons during development between Ler-0, Col-0 and Ws-0 ecotypes [3], quantitative trait loci (QTL) analysis of leaf and floral organ size of 162 recombinant inbred lines (RIL) from a reciprocal cross between Ler and Cvi [4] and seed size of the *iku*2-1, *fis*2-1, *arf*2, *pAP*1*::ARF*2 mutants and Col-0 and Ler-0 ecotypes [5]. Examples of the latter are the effects of drought, low temperature and differing levels of UV-B on chlorophyll-fluorescence on growth [6] and the natural variability of 23 accessions in response to nitrogen [7]. Third, *Arabidopsis* is a widely used model system with sophisticated genetic and genomic resources [8] available for dissecting biological processes. Forward genetic approaches have been used to study mutants with strong phenotypic effects providing insight into the underlying molecular functions. While this approach is extremely useful as a research tool, commercial plant breeding often requires exploitation of continuous variation. Analysis of continuous variation in breeding populations is more demanding, but effective automation of the phenotype measurements would have huge advantages for crop improvement and food security. *Arabidopsis* is also a good model for studying continuous variation, with the advantage of thoroughly investigated genomics [9], [10]. The native range is North-Western Eurasia and it has recently colonised other parts of the world during the Columbian Exchange [11]. Local populations have often diverged, to a degree depending on factors such as time of separation and differential selection. The species is therefore a useful model to study natural variation, its underlying genetic basis and its consequences.

Natural variants provide material for studying genome evolution and the genetic dissection of complex traits. The *Arabidopsis* lines used here are the inbred parental lines of a MAGIC mapping population, which have been selected from a wide range of locations and cover a range of genetic diversity [12]. The MAGIC population contains several hundred recombinant inbred lines (RILs) descended from the 19 founder lines, via a series of defined intercrosses. These RILs and the 19 founders have been genotyped and previously scored for development-related traits such as flowering time [1].

**Figure 1:**
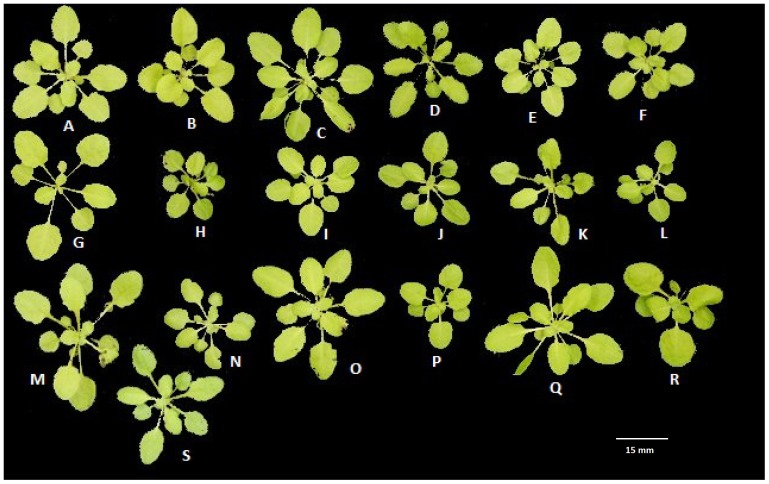
Images of representative plants of the 19 parental lines [1] at 28 DAS. (A) EDi-0, (B) Can-0, (C) WS-0, (D) Hi-0, (E) Zu-0, (F) Po-0, (G) Sf-2, (H) Wil-2, (I) 0y-0, (J) Kn-0, (K) Ct-1, (L) Wu-0, (M) Tsu-0, (N) Col-0, (O) Rsch-4, (P) No-0, (Q) Bur-0, (R) Ler-0 and (S) Mt-0.

High throughput methods that are suitable for measuring continuous variation have been applied to *Arabidopsis*. For example, a single time point study characterised *Arabidopsis* plants grown in vitro by analysing rosettes, leaves and leaf cells in 111 mutants and three wild-type accessions [13]. In another study, rosette areas of ecotypes were analysed across developmental stages using an automated phenotyping pipeline [14]. Responses to soil water deficit of natural accessions have been reported from the PHENOPSIS platform [15]. Similarly, GROWSCREEN investigated growth potential of starch-free mutants of *Arabidopsis* [16]. These approaches often use non-destructive image analysis as an element of growth measurements. However, for complex biological objects there is no generally accepted method of parameterising shapes, which makes quantification of change and determination of significance of differences difficult, particularly for characters subject to continuous variation. In *Arabidopsis* some aspects, such as leaf shape [17], [18] have been studied in detail, but usually by destructive methods. Also, the technique used (principal component analysis of points on the leaf margin) is difficult to apply to overlapping composite structures that change in shape as the plant grows, as in *Arabidopsis* rosettes (Figure 1).

Using the 19 parental lines as a test population, we investigate the ability of a range of morphological descriptors developed for image analysis [19] to discriminate rosette images, an exercise not previously reported for *Arabidopsis*. We used machine-vision methods to analyse shape descriptors extracted from segmented images of rosettes [20] and provided a wider range of parameters with definitions of their calculations than previously reported. This method does not require any specialised equipment for image capture and is therefore generally applicable. Two different commercially available software packages were evaluated and used to extract descriptors. This allowed us to compare the accessions according to their phenotypic diversity and to address the following questions: 1) Are shape descriptors time-dependent, ecotype-dependent or both? 2) What are the most important descriptors of rosette shape among the set tested? 3) Do shape descriptors differ between the ecotypes? 4) If shape descriptors change with time, is the direction and amount of change similar in all ecotypes? We show that shape descriptors can be used to parameterise and determine significant differences between rosette shapes of *Arabidopsis* lines and we discuss the potentially wider role of these characters. We also compare our data with that of a previous study in different media and at a single time point [13], to assess the generalizable ability of these results This provides a cost-effective, scalable method to parameterise shape that can be used non-destructively on whole plants.

## Materials and Methods

### Plant material

Seeds of 19 genetically contrasting but individually uniform ecotypes [12] (EDi-0, Can-0, WS-0, Hi-0, Zu-0, Po-0, Sf-2, Wil-2, Oy-0, Kn-0, Ct-1, Wu-0, Tsu-0, Col-0, Rsch-4, No-0, Bur-0, Ler-0, Mt-0) of *Arabidopsis thaliana* were sown on moist Levington F2 compost (Scotts UK Professional, Bamford, Suffolk, UK) in 24 pot (each pot 51 by 47 by 47 mm) trays kept at 4C for one week before moving to a controlled environment room (CER) at 23°C day/20°C night, day/night (8/16 h) and 110 µmol m^−2^ s^−1^ PPFD (Sylvania VHO fluorescent tubes). These conditions were chosen to be reasonably similar to the conditions recommended in a laboratory manual for the growth of *Arabidopsis* (16–25°C, 120–150 µmol m^−2^ s^−1^ PPFD), with a day length of less than 12 hours to avoid acceleration of reproductive development [21]. Twelve replicates were grown per ecotype and trays were watered with a conventional watering can. Trays were also moved and rotated regularly to randomise local environmental variables and watering. No further nutrients were supplied during the course of the experiment.

### Data acquisition

Photographs of rosettes were taken using a Panasonic DMC-G1 camera mounted horizontally on a tripod at a resolution of 3000×4000 pixels on 17, 22, 25, 28 and 30 days after stratification (DAS), t_1_ to t_5_ respectively. Images were taken between 10.20 am and 11.20 am in the growth chamber where light and temperature were kept constant. A composite image of typical plants on 28 DAS is shown in Figure 1. Using an internal length reference, a scaling factor was calculated (average 7.31 pixels per mm SEM 0.104) and applied to each image to compensate for any slight variation in the position of the tray relative to the camera and to convert pixel-based measurements to dimensions to facilitate comparisons with other work. All measurements were made in the early stages of plant development, before significant floral development, when it was reasonable to analyse the rosette as a two dimensional structure.

### Image analysis

**Figure 2:**
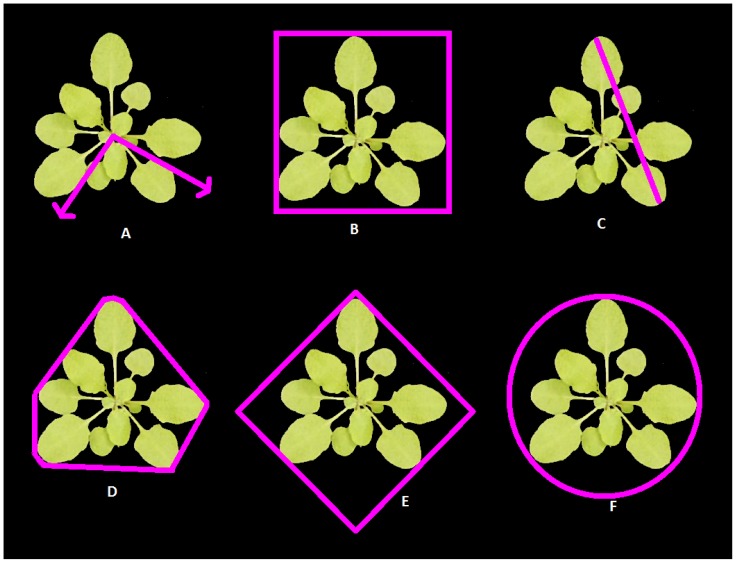
Example features extracted from one plant of EDi-0. (A) normsmallpax and lormlargepax, (B) vrectsizex and vrectsizey, (C) maxdiam, (D) outline of convex hull, from which convhullcirc and convhullarea are determined (E) minrectarea, (F) mincirclediam.

Image processing was performed using LemnaGrid software from LemnaTec [22] in the following sequence: 1) Nearest neighbour foreground/background colour separation was used to classify pixels. Two sets of colour intensities, corresponding to foreground (target) and to background (non target) are selected. RGB pixels matching selected intensities are mapped into the image. A search around mapped pixels is performed to identify pixels with similar intensities that might be part of the foreground/background regions. Once the search is performed, the image is converted to binary, where 1 is the target (plant) and 0 the background (compost, tray, etc). 2) Morphological techniques were then applied to deal with pixels incorrectly classified [19]. First, morphological erosion was applied to remove small and isolated pixel regions incorrectly classified as plant. Second, morphological dilation was applied to correct for those pixels located in the border of the images that were incorrectly classified as background. 3) A final filter operation used the area of the pots to mark approximately the region occupied by each plant in the tray, and foreground pixels outside this region were deleted. Once the images were segmented, 20 image features (Table 1 shows the list of features - descriptors) describing the geometry, shape and size of rosettes were extracted. Some of these features are illustrated in Figure 2 (an example set is shown in Figure S1). Table S2 shows the values corresponding to the descriptors highlighted in Figure S2. In Methods S2 further details of the specific calculations used, their labels in the LemnaTec software, their equivalents in Matlab [23] and the complete data table of image features are provided.

**Table 1:** Shape descriptors extracted from segmented *Arabidopsis* rosettes.

Id^1^	Name	Description	Group^2^	Trans^3^	Unit
1	Mincirclediam	Diameter of the smallest circle the rosette would pass through without touching.	A	log_e_	mm
2	Normsmallpax	Ratio of the Sum of distances of all pixels from smaller axis in direction of the larger axis normalised to rosette area	N		Ratio
3	Normlargepax	Ratio of the Sum of distances of all pixels from the larger axis in direction of the smaller axis. normalised to rosette area	N		Ratio
4	Minrectarea	Area of smallest rectangle of any orientation covering rosette	A	log_e_	mm^2^
5	Mindistcenbdy	The distance between the centroid and the nearest point on the rosette boundary	N		mm
6	Vrectsizey	Height of the smallest vertical rectangle covering the rosette.	A	log_e_	mm
7	Vrectsizex	Width of the smallest vertical rectangle covering the rosette	A	log_e_	mm
8	Compactness	Rosette Area/Conhullarea	N		Ratio
9	Normrotmo	Moment of inertia of rosette around centroid	N		Ratio
10	Area	Area of rosette	A	log_e_	mm^2^
11	Paxratio	Normlargepax/Normsmallpax	N		Ratio
12	Circumference	Perimeter of rosette excluding holes	A	log_e_	mm
13	Excentricity	Ratio of Normlargepax to Normsmallpax	N		Ratio
14	Maxdiam	Maximum distance between two points on the rosette boundary	A	log_e_	mm
15	Roundness	Circumference^2^/Area	N		Ratio
16	Bdryround	Bdrycount^2^/area.	N	log_e_	Ratio
17	Bdrycount	Boundary of rosette including perimeter and holes	A	log_e_	mm
18	Bdrytoarearatio	Bdrycount/Area	N		mm^−1^
19	Conhullcirc	Length of convex hull, the line with no concave sections surrounding the rosette	A	log_e_	mm
20	Conhullarea	Area included in Conhullcirc	A	log_e_	mm^2^

1 id is index to descriptors used in Figure 5

2 Indicates whether descriptor is in the Area (A) or non-Area group (N)

3 Indicates if data were transformed using a loge scale.

The features used to describe shape can be grouped into two classes, those with a simple meaning and definition and those whose interpretation is more complex. For example, comparing ‘Area’ and ‘Roundness’: ‘Area’ is the pale green region comprising the rosette within the rectangle in Figure 2; ‘Roundness’, indicating circularity of the object, is more complex as it is given by the ratio of Circumference^2^ to Area, where ‘Circumference’ is the total length of the lines forming the outside of the rosette. Simple and complex features are necessary to give a complete description of the shape of the object and will be used to quantify variation between shapes of parental lines. The relationship between the shape descriptors over a range of rosette shapes is illustrated in Figure S1. In addition, Relative Rosette Area Growth Rate (RRAGR) was calculated as:
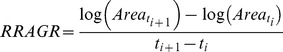
(1)where Area
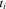
is the rosette area at time *t_i_*, with *t_i_* as defined above. RRAGR were plotted and analysed at the mean time for RRAGR calculation.

### Data processing and analysis

The descriptors were loaded into a dataset and after Shapiro normality tests appropriate log_e_ transformation were applied when necessary to produce normality (Table 1). The Bonferroni Outlier Test was applied to identify and remove outliers that could bias the results. A linear mixed effect (lme) model for repeated measurements [24] was fitted for each character (Eq. 2). This modelling approach was favoured against other alternatives as it is appropriate when dealing with time series, when the variances of the observations are unequal or when there is a degree of correlation between measurements. 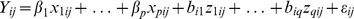
(2)

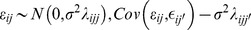
Where *Y_ij_* is the response variable for the *j*th of n*_i_* observations in the *i*th of *M* ecotypes

*x_1ij_, …, x_pij_* are the fixed-effect regressors for observation *j* in group *i*

*bi_1_, …, b_iq_* are the random-effect coefficients for group *i, i = 1, .., q* number of groups

*z_1ij_, …, z_qij_* are the random-effect regressors

*β_i_, …, β_p_* are the fixed-effect coefficients, *i = 1, .., p* number of effects

ε*_ij_* is the error for observation *j* in group *i*

σ^2^λ*_ijj’k_* are the covariances between errors in group *i*. If the observations in a group represent longitudinal data on a single individual (e.g. observations collected over time), then the structure of the λ's is specified to capture autocorrelation among the errors.

The post-hoc Tukey test was applied to perform pairwise analysis over the fitted models P-values were adjusted using the Bonferroni multiple test correction (P<0.05).

ReliefF [27] and Principal Component Analysis (PCA), two contrasting methods of determining the relative importance of multivariate characters were used to compare variability of shape descriptors. ReliefF estimates the quality of attributes in classification problems with strong interdependencies and PCA (with variables scaled to zero mean and unit variance) was used to determine underlying grouping variables which summarise variability in the data. All statistical analysis were carried out using the R [25] and the Weka [26] packages.

## Results

**Figure 3:**
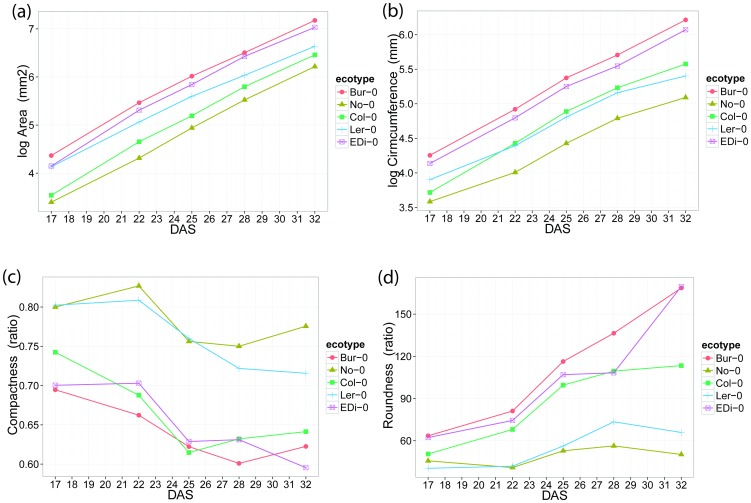
Time course for selected genotypes from statistical model for (A) log_e_ Area (B) log_e_ Circumference (C) Compactness and (D) Roundness.

The evaluation of time courses of ecotype shape descriptors proceeded in three stages: Firstly, differences in shape parameters between the ecotypes (an ecotype effect) were determined. Secondly, changes with time were across all ecotypes (a time effect) were assessed, and thirdly differences in the changes with time between ecotypes (an interaction effect e.g. time x ecotype) were checked. Inspection of plots of the shape descriptors across time and ecotype were used to establish general characteristics of the data (see Figure 3 for an example plots and the full set in Figure S2). They also helped design statistical models for each descriptor. These plots clearly fall into two groups (Table 1), one whose time course was continually rising similar to that of Area (Area group) and those whose time courses were much more variable in slope and direction (the NonArea group). More details of these groups are in the ‘Analysis of descriptors over time’ section below. The Area Group characters have a quadratic time course, suggesting that a quadratic effect of time should be included in any statistical model. However, we recognise this is a simplification of the complete growth curve, possibly sigmoid, for which we have partial data only. The same plots also showed that shape descriptors, for both groups: a) changed over time (not horizontal lines across the time), suggesting time dependency; b) were significantly different between ecotypes (lines touched or overlapped), suggesting ecotype dependency and c) were time and ecotype dependent.

As the experiment was a split-plot design replicated in time (to asses changes over time), a linear mixed effect model that took in consideration the effects of the repeated measurements design and the variance and covariance of each shape descriptor was used. The model included a random intercept for each plant, an ecotype effect, a time effect, and an ecotype-time interaction effect. In addition for the Area group DAS squared was added to the model to allow for the quadratic trend with time. Eq. 3 is the model of Compactness for observation *j* in the group *i* at *t* time point. Eq. 4 is the model of Area for observation *j* in the group *i* at *t* time point.
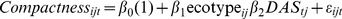
(3)

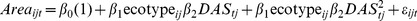
(4)


Results of the lme models for each shape descriptor confirmed that (a) there was a significant time effect (P<0.05), suggesting time dependency, (b) there was a significant ecotype effect (P<0.05), suggesting ecotype dependency and (c) there was a significant interaction effect (P<0.05) between time and ecotype. Plots of residuals showed normally distributed errors with homogenous variances providing an indication of the goodness of fit of the model describing each shape descriptor.

Figure **3** shows time courses from fitted models for ‘Area’, ‘Circumference’, ‘Compactness’ and ‘Roundness’ for selected ecotypes.

**Figure 4:**
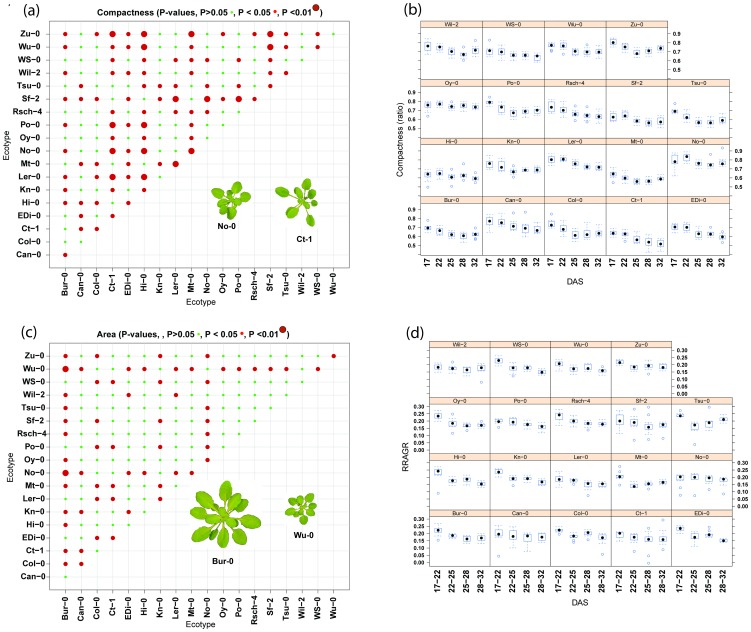
Multiple comparisons of Compactness and Area between ecotypes. Scatter plots showing significant (P>0.05•, P<0.05 •, P<0.01•, Post-hoc Tukey test between ecotypes) differences between ecotypes for (A) ‘Compactness’ and (C) ‘Area’ and boxplots for (B) ‘Compactness’ and (D) Area over time. Images have been added to (A) and (B) to show range of ‘Compactness’ and ‘Area’.

To determine over which time steps the differences in the shape parameters were significant across all ecotypes, a pairwise Tukey test over the lme model was applied between DAS. ‘Normsmallpax’, ‘Normlargepax’ and ‘Normrotmo’ showed significant differences only between t_1_ and t_2_. ‘Mindistcenbdy’ showed significant differences only between t_2_ and t_3_. ‘Excentricity’ showed significant differences only between t_3_ and t_4_. ‘Compactness’ is relatively constant with time and generally becomes stable after t_2_ suggesting that ‘Compactness’ is defined early in development. Images of rosettes (Figure 4A) contrast the low ‘Compactness’ in Ct-1 due to long petioles with the greater ‘Compactness’ of No-0 with its much more closely spaced leaves. Other descriptors such as ‘RRAGR’ show most significant (P<0.01) differences between the pairs ‘Tsu-0/Wil-2, ‘Tsu-0/No-0’ and ‘Tsu-0/Ler-0’ (Figure S7). Differences between all-time points were significant (P<0.05). ‘Area’ was significantly different between all time steps (P<0.05), as would be expected for a size parameter of growing plants (Figure 4). Between ecotypes ‘Area’, showed the most significant differences (P<0.01) between the pairs ‘No-0/Bur-0’, ‘Bur-0/Wu-0’ and ‘EDi-0/No-0’. Images of Bur-0 and Wu-0 have been added to Figure 4C to illustrate the greater size of the rosette in Bur-0 than Wu-0. Figures 4B and 4D use box plots to illustrate the changes in ‘Compactness’ and ‘RRAGR, and the variation between replicates, for each ecotype across time.

**Figure 5:**
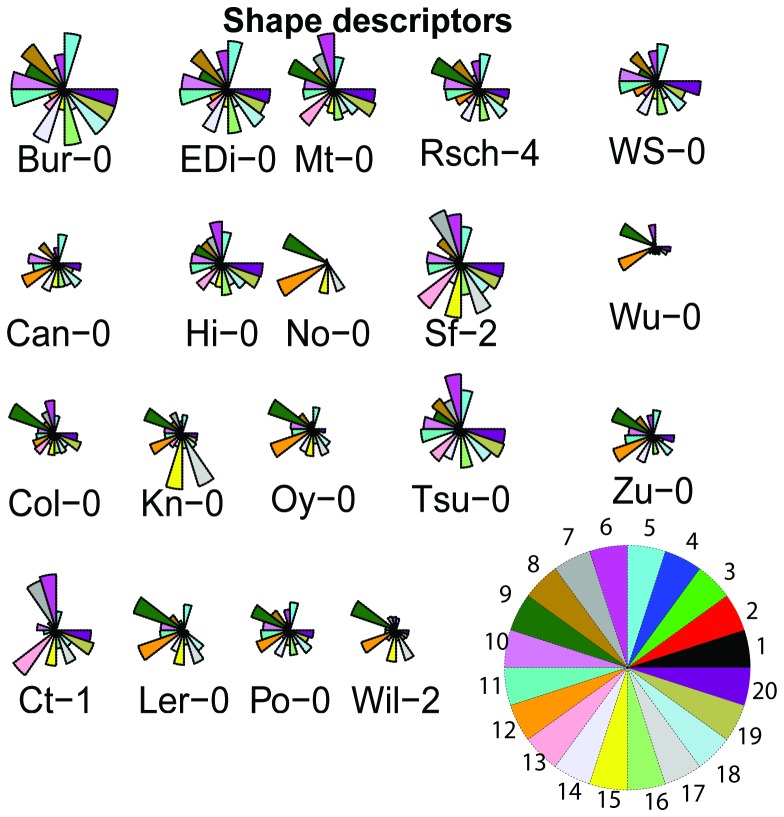
Shape descriptor plots of ecotype's feature profile. Radar plot of 20 shape features for each ecotype. Plots are average of 12 replicates over five time points. Variable assignment key for star plot is located at the bottom right corner. Each star plot or segment diagram represents one row of the input data. Variables (columns) start on the right and wind counter clockwise around the circle. The size of the (scaled) column is shown by the distance from the centre to the point on the star or the radius of the segment representing the variable. The columns of the data matrix are scaled independently so that the maximum value in each column is 1 and the minimum is 0. Segment numbers corresponding to descriptors are defined in Table 1.

Having established that shape descriptors changed at different rates through time and that this pattern of change differed between ecotypes, we asked whether ecotypes could be grouped on the basis of their shape parameters. Visual inspection of radar plots for each ecotype (Figure 5) indicate that Bur-0 and EDi-0, No-0 and Wu-0, Po-0 and Can-0 share a similar overall pattern of parameters, while Po-0 and Can-0 are very similar in terms of ‘Compactness’ and ‘Area’. The plots also show that No-0 and Ler-0 are both quite compact but Ler-0 has a greater rosette ‘Area’. The radar plot is an excellent way to rapidly identify groups that are common at a given descriptor.

### Feature selection

**Figure 6:**
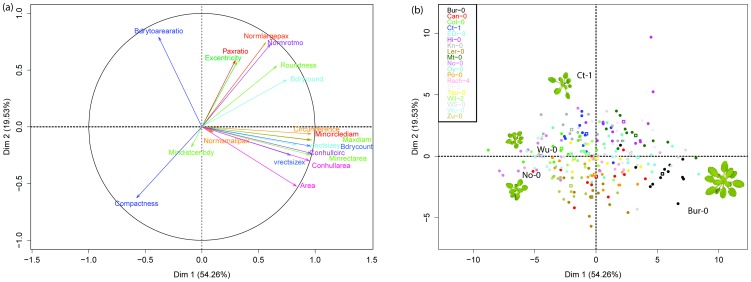
Principal Component Analysis. (A) Variable loadings for first and second principal components, 17 DAS and (B) Analysis by ecotype over first and second principal components, 17 DAS. Significance level is given by the size of the squares, the smaller the square the more significantly different (P<0.05). (C) Contrast in ‘Area’ between ‘Bur-0’ and ‘Wu-0’ and Contrast in ‘Compactness’ between ‘Ct-1’ and ‘No-0’) and where both ecotypes have similar Area as demonstrated in plot (A).

PCA was performed per time point to identify meaningful underlying variables and whether they show the same pattern across time. At 17 DAS 54.26% of the variation was captured by the first principal component and 19.53% by the second, (Figure 6A). PCA plots for 22, 25, 28 and 32 DAS are shown in Figure S3. In summary, the major contributors to PC1 were ‘Maxdiam’, ‘Conhullcirc’, ‘Circumference’, ‘Mincirclediam’, ‘Bdrycount’, ‘Minrectarea’ and ‘Conhullarea’. The major contributors of PC2 were ‘Bdrytoarearatio’, ‘Normlargepax’, ‘normrotmo’ and ‘Compactness’. PC3 captured 14.3% of the variation and the major contributors were: ‘Normsmallpax’, ‘Paxratio’ and ‘Excentricity’. Variation captured in further components was: PC4: 5.14%, PC5: 2.68%, PC6: 2.04% and PC7 to PC20's summed was 2.04%. Interestingly, the major contributor of PC4 was ‘Mindistcenbdy’ (contributing 84%) suggesting a more detailed look to this descriptor in future analysis. Figures S4A–D show standard contributions for each descriptor.

PCA of all observations labelled by time showed that there was more variability later than earlier in growth (Figure S5).

Further, we applied the ReliefF method for attribute estimation in the regression to reduce the dimensionality of the data. This method assesses a set of features in a dataset and ranks them according to their power to accurately differentiate classes (i.e. ecotypes) in the dataset. The test ranked ‘Compactness’, ‘Normrotmo’ and ‘Normlargepax’ in first, second and third place and ‘Conhullcirc’ in last, confirming that ‘Compactness’ is an important discriminatory descriptor of rosette shape. This analysis also shows that ‘Area’ and ‘Area-related’ descriptors are not good discriminators of ecotypes. The Area plots in Figure S5 shows a similar trend for each ecotype.

### Growth patterns of ecotypes

To evaluate the extent to which the patterns of growth were influenced by the time, data points classified by time were added as supplementary information to the PCA analysis. Results in Figure S5 show data points are located at the centre of the plot at an early stage and become scattered around at a later stages which suggest that plants showed more variability as the get bigger. In addition to time, ecotype variation was also analysed, Figure 6 shows results. Some points to highlight from this analysis are: there were significant differences (P<0.05) between the ecotype's shapes (given by the size of the square, the smaller the square the more significant). Bur-0, EDi-0 and WS-0 are on the same PCA dimensions suggesting similarities between them in relation to shape. This result is consistent to what is shown in the radar plots of Figure 5 where Bur-0 and EDi-0 show similar patterns. Ct-1 shows similarities with the other ecotypes at 17 DAS but soon its data points are scatter along dimension 2 (Figure S6A–D). Data points of Oy-0, Ler-0, and Zu-0 are on the same coordinates of ‘Compactness’ (Figure S6A–D) which suggests that these three ecotypes are correlated in ‘Compactness’. To help in the analysis, rosette images of some ecotypes were superimposed on the plot. For example, data points corresponding to No-0 are on the coordinates of ‘Compactness’ and data points corresponding to Ct-1 are at the opposite site. This data organization suggest low correlation in ‘Compactness’ which is confirmed by the rosettes images of Ct-1 and No-0. Similarly, Bur-0's data points are on the coordinates of ‘Area’ and Wu-0 are at the opposite site, suggesting low correlation in ‘Area’ which is confirmed by the rosettes images of Bur-0 Wu-0.

### Analysis of descriptors over time

Phenotypic traits can be classified as static or dynamic. Static traits are often complex characteristics that tend to be measured at a single point in time (for example, yield), whereas dynamic traits (growth and other spatiotemporal changes) change with and can reveal different trajectories to similar end points [28], [29]. We analysed the trajectory of each descriptor over time and found two main patterns. The first pattern, the ‘Area’ group, defined above (Table 1) contained descriptors with a continually increasing plant size component as seen for ‘Area’ (Figure 3 and S2). The trajectories appeared to be parallel between ecotypes with a time displacement, Bur-0 having the greatest and No-0 the smallest ‘Area’ at the last measurement. Comparisons indicate that the increases in ‘Area’ are attributable to growth rather than changes in plant shape as would be the case, for example, if petiole elongation played a major role. Areas were already different when seedlings were first imaged and these differences were largely maintained during the period of the experiment. Increased ‘Area’ at first measurement may be the result of increased seed size at sowing as seeds of Bur-0 have been reported as larger than those of Col-0 and Ler-0 [5], However, seed areas (a measure of seed size) were not correlated with rosette area from multiple replicates of each ecotype and from two batches of seeds from the same source as used here.

**Figure 7:**
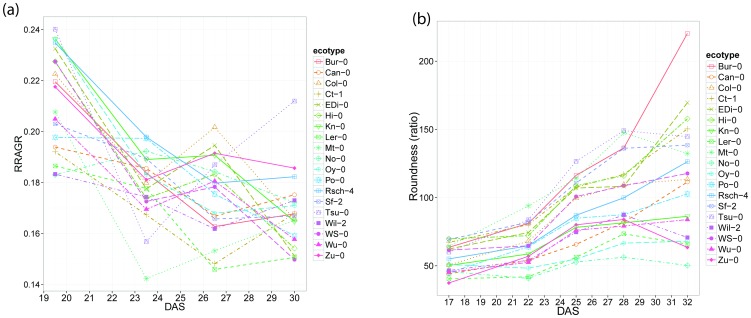
Shape descriptors over time. (A) Relative Rosette Area Growth Rate Over Time (RRAGR), (B) Compactness.

The second group, the NonArea group, (Table 1) show variable patterns of change (Figures 7B and S2). This group includes descriptors of shape that relate to how the area is distributed to form the resultant rosette shape. Rsch-4 had the greatest ‘Mindistcenbdy’ and Can-0 the lowest (Figure S2). Within the overall pattern of compactness described above, there were noticeable differences between ecotypes with No-0 decreasing less than Ct-1 (Figure 7B). ‘Paxratio’ generally decreases over time with Sf-0 having the lowest and Bur-0 the highest. However, Ct-1 shows a different pattern, increasing moderately until t_4_ and thereafter decreasing moderately. ‘Roundness’ increases over time with Bur-0 being the highest and No-0 the lowest. Bur-0 becomes rounder at every time step, which contrasts with all the other ecotypes. ‘Bdryround’ increases over time with Bur-0 increasing fastest and Ler-0 increasing more slowly. ‘Excentricity’ decreases over time with Bur-0 being the most rapid and Sf-2 the slowest. ‘Bdrytoarearatio’ is defined as the length of the outline divided by the area, and this relationship would be expected decrease as the plant grows on purely mathematical grounds. The rate of decline in the ‘Bdrytoarearatio’ was greater in Ler-0 than in Ct-1, the two ecotypes display extreme values for this parameter. See distribution of shape descriptors over time in Figure S2.

### Comparisons with a previous study

One of the principal problems in using a new approach such as the morphological descriptors used here is determining if the descriptors are universal To investigate this we have compared our results with a similar approach used previously [13]. Whilst both approaches were used on similar material, i.e. the rosettes of young *Arabidopsis* plants, there were important differences in the range of material used and cultivation (summarised in Table S3) and the previous data was collected at only one time point. Some comparable descriptors were present in both studies although in detail some were calculated slightly differently (see Table S4). PCA of the previous descriptors on our dataset (Figure S8A) produced a very similar distribution of principal components 1 and 2 to that in [13]. Although PC1 and PC2 accounted for more of the variation (82 and 13% for PCs 1 and 2 in our data compared with 78 and 11% respectively in [13]), probably an effect of the smaller number of variables used in our PCA. As in [13] most of the Correlations between area related parameters: Rosette area (RA), Rosette Perimeter (RP), Ellipse Area (EA), Ellipse Perimeter (EP) and Max Feret's diameter (RXF), were positive (Table S5, Figure S8B). Because of the wider range of the parameters due to the greater time for growth, these parameters appear to be more closely related than in [13]. Also the smaller number of ecotypes in our data set compared with the much larger number of lines in [13] may have resulted in the closer relationships in our data. As before the relations of the area based parameters with Rosette compactness (RC) were less well defined. Thus our results are largely in agreement with [13] but extend the approach to multiple time points, Also, our study took into account the repeated measurements and used statistical models to determine significant differences between ecotypes, time and their interaction, for each descriptor. We also reported more descriptors and define how each of was calculated to allow for other readers to repeat the analysis.

## Discussion

Rosette shape and size are attractive traits on which to develop and evaluate automated approaches for objective machine-assisted plant phenotyping. The change in shape and size can describe how the plant (or crop) covers the ground surface, affecting traits such as photosynthetic potential and canopy closure. The latter affects a crop's ability to suppress competition [30] and, in dry climates, may affect soil water conservation. In *Arabidopsis*, rosettes lie approximately flat across the ground so simple 2D photography can acquire most of the relevant information.

Despite being intrinsically amenable to automated acquisition and analysis, there are few detailed studies of rosette shape surveying natural variation amongst a range of accessions as in the MAGIC plants here. In this study, aimed at developing non-destructive objective methods to monitor plant growth, we have used simple computational methods to process, analyse and compare the rosettes of 19 ecotypes of *Arabidopsis*. Images were taken at successive times to capture the patterns of growth, segmented to separate rosettes from background and features describing rosette shapes were extracted and analysed. Shape descriptors were compared to establish whether differences between ecotypes were significant. An advantage of this approach is that the descriptors used are either independent of plant orientation, or are calculated taking into account the principal axis of the rosette. The continuous and complex variation in rosette shape has been reduced to numerical descriptors, which was here examined for differences using established statistical techniques. Significant differences for the descriptors have been established between ecotypes (Figure 6) and two independent presentations revealed a similar pattern of relationships between the ecotypes (Figure 5). These two analyses suggested that shape descriptors were time and ecotype dependent because the time effect was significant (P<0.05), the ecotype effect was significant (P<0.05) and the interaction between time and ecotype was also significant (P<0.05). To confirm which of those ecotypes with apparently different time courses on initial visual inspection (Figure S2) were significantly different, post-hoc tests between pairs of ecotypes were used. Furthermore, the RRAGR interaction plots (Figure 7A) and the post-hoc tests suggested that descriptors such as ‘Area’ do not change with the same direction and amount in all ecotypes (Figure S7). Those changes could not be attributed to seed size as seed size was not correlated with rosette area.

In addition to the statistical results, PCA and machine learning analyses (such as the ReliefF method used here) concluded that ‘Area’ and Compactness were among a set of candidates that could be used for rosette classification. Other studies have also demonstrated that ‘Area’ accounted for the highest variability [13]. As most of the shape descriptors used ‘Area’ as parameter, perhaps other descriptors associated with ‘Area’ could be used for classification, for example RRAGR (Figure S3E). Note that PCA plots (Figure 6 and Figure S3A-D) show Area as clustered with a number of other shape descriptors and not completely isolated from the rest.

While this paper describes the application of an initial set of unbiased quantitative characters to describe variation in rosette shape of *Arabidopsis*, other shape descriptors are available. Some of these may be more robust to differences in viewpoint and scale and some assess other aspects of shape that are not included here [31]. We restricted our study to two dimensions, an obvious over-simplification. Rosettes are significantly variable in 3-D; capturing the 3^rd^ dimension requires more sophisticated image vision tools. Subsequent floral development is highly dynamic in all three spatial dimensions and requires additional descriptors. Furthermore, physiological processes contribute to colour variation that is super-imposed on morphological variation. Some techniques (such as LemnaTec 2.5D) provide a very rough proxy, but most other currently used methods are in effect multiple 2-D analyses, and the same pipeline for plant management, image processing and data analysis could be used. It remains a very significant challenge to screen populations in 3-D but 3D models have been produced from rotary imaging [32] or laser scanning [33] of single plants. Thus, the development of an optimal set of descriptors suitable for capturing general traits remains to be completed and is partly dependent on the imaging technology available.

A widespread and probably under-reported problem in plant biology is that, in spite of very careful control of growth conditions, it seems remarkably difficult to grow plants in a reproducible manner between laboratories [34]. As the measurements used in this report are non-destructive and rapid they provide a completely unbiased means of measuring growth responses for given genotypes across different locales, times and environments. Essentially this would use genetically defined plants as an environmental monitor with objectively acquired plant morphological descriptors as the readout, allowing results to be normalised. This could replace manual, often subjective, observations and allow improved comparisons between experiments across workers and laboratories. Also the information contained in the shape descriptors, together with the existing descriptions of leaf shape [17], [35], should allow reconstruction of a representation of the average plant with its statistically described range under given conditions. These reconstructions could then be developed for plants grown under a range of conditions and will allow a thorough cataloguing of environmental responses that can be used to diagnose those features of the environment that the plant is responding to. In that regard, we compared our results against the Perez-Perez study and found important similarities.

Both commercial (e.g Matlab, LemnaTec) and freeware (i.e. ImageJ [36], Octave [37] or Scilab [38]) packages are capable of extracting and analysing appropriate descriptors are readily available. However, in some commercial packages, the precise mathematical identity of the descriptor is obscure and can change without notification. This reduces their value to the general research community. We include a list of equations (Table S1) defining the majority of descriptors as used in version 2.1.0.8645 of LemnaTec. These equations were written in the Matlab environment to provide another way to calculate the shape descriptors. The definition of variables are in the files (Methods S1 and S2); variable names used to store final results of calculations in the Matlab program on a binary image are given in Table S6 to facilitate comparison of results using other software. It is also important to convert pixel-based values to appropriate dimensions to facilitate comparisons of images takes on different cameras with different optics and different distances. We suggest that all such descriptors should be clearly described by software purveyors, so that they can be used in the creation of technology-standard ontologies.

In general terms, the same principles should be applicable to more complex plant architectures, including later developmental stages of *Arabidopsis* as well as to crops and grasses of economic importance. The challenge will be to process and characterise large amounts of 2- and 3-D image and physiological data taken from automated capture systems handling large numbers of plants. Therefore, phenotype-genotype relationships for plant improvement can benefit hugely, and descriptions of shape phenotypes will become as accessible to analyses as currently available for genotypes. Here we have provided equivalent analysis in open source software and have provided the equations the calculation image descriptors which are closer to the ideal for reproducible computational research [39].

## Supporting Information

Figure S1 **Illustration of variation in the shapes of Arabidopsis rosettes and of descriptors (as defined in** **Tables 1** **and S1, with values given in table S2.** Rows in order from top: Segmented image; Normsmallpax and Normlargepax; Vrectsizex and Vrectsizey; Maxdiam; convex hull from which Conhullarea and Conhullcirc are derived; Rectangle from which Minrectarea is determined and (bottom row) the circle from which Mincirclediam is determined. The rosettes in the image are artificial, they correspond to one Arabidopsis rosette manually modified to demonstrate the meaning of the descriptors.
(TIF)

Figure S2 **Time courses.** Shape and size descriptors and area growth rate for the 19 Ecotypes. Values are averages per ecotype at each time point.
(ZIP)

Figure S3 **PCA at successive time points.** (A) 22 DAS, (B) 25 DAS, (C) 25 DAS and (D) across all-time series.
(ZIP)

Figure S4 **Relative contributions of all variables to principal components 1 to 5 for analysed separately 22, 25 28 and 32 DAS.** (For 17 DAS see Figure 6).
(ZIP)

Figure S5 **Principal components 1 and 2 from PCA combined over all ecotypes and times with points labelled by time.**
(TIF)

Figure S6 **PCA For each time and grouped by ecotype.** (A) 22 DAS, (B) 25 DAS, (C) 28 DAS and (D) 32 DAS. Small squares around the ecotypes show significant differences (P<0.05) between ecotypes. The smaller the square the more significant the difference.
(ZIP)

Figure S7 **Multiple comparison of RRAGR.** Scatter plots showing significant (P>0.05_•_, P<0.05 •, P<0.01_•_, Post-hoc Tukey test between ecotypes).
(TIF)

Figure S8 **Results from analysis of descriptors here also used in** [13] **.** (A) PCA and (B) Scatter plot showing relations between seven descriptors.
(TIF)

Table S1 **Equivalence of names provided by LemnaTec software.**
(DOCX)

Table S2 **Values of features extracted from rosettes illustrated in Figure S1 used to show variation in rosette descriptors with id of descriptor as in** **Table 1** **.**
(DOCX)

Table S3 **Comparison of experiments between Perez-Perez and Camargo studies.**
(DOCX)

Table S4 **Correspondence of shape descriptors in Perez-Perez and this study.**
(DOCX)

Table S5 **Correlation coefficient between each descriptor and principal component one.**
(DOCX)

Table S6 **Variable names used to store final results of calculations in the Matlab program on a binary image stored as bw.** Also scale factor (sf) applied to convert units from pixel based values to measurements (where value is not a ratio then no entry).
(DOCX)

Methods S1 **Description of calculations of shape parameters.**
(DOCX)

Methods S2 **Compressed file contains a number of files.** 1) Raw data. Descriptors extracted from each segmented image, 2) R scripts used to handle and analyse data, 3) Matlab scripts used as another way to extract the 20 shape descriptors used in this analysis, 4) Example image suitable for processing by the Matlab script and 5) File descriptions, summary of files in this zip file.
(ZIP)

## References

[1] Kover PX, Valdar W, Trakalo J, Scarcelli N, Ehrenreich IM, et al (2009) A Multiparent Advanced Generation Inter-Cross to Fine-Map Quantitative Traits in *Arabidopsis thaliana* . PLoS Genet 5: e1000551. 19593375 10.1371/journal.pgen.1000551 PMC2700969

[2] Furbank RT, Tester M (2011) Phenomics – technologies to relieve the phenotyping bottleneck. Trends in Plant Science 16: 635–644. 22074787 10.1016/j.tplants.2011.09.005

[3] Passardi F, Dobias J, Valério L, Guimil S, Penel C, et al (2007) Morphological and physiological traits of three major *Arabidopsis thaliana* accessions. Journal of Plant Physiology 164: 980–992. 16904792 10.1016/j.jplph.2006.06.008

[4] Juenger T, Pérez-Pérez JM, Bernal S, Micol JL (2005) Quantitative trait loci mapping of floral and leaf morphology traits in *Arabidopsis thaliana*: evidence for modular genetic architecture. Evolution & Development 7: 259–271. 15876198 10.1111/j.1525-142X.2005.05028.x

[5] Herridge R, Day R, Baldwin S, Macknight R (2011) Rapid analysis of seed size in *Arabidopsis* for mutant and QTL discovery. Plant Methods 7: 3. 21303553 10.1186/1746-4811-7-3 PMC3046896

[6] Jansen M, Gilmer F, Biskup B, Nagel KA, Rascher U, et al (2009) Simultaneous phenotyping of leaf growth and chlorophyll fluorescence via GROWSCREEN FLUORO allows detection of stress tolerance in *Arabidopsis thaliana* and other rosette plants. Functional Plant Biology 36: 902–914. 10.1071/FP09095 32688701

[7] Ikram S, Bedu M, Daniel-Vedele F, Chaillou S, Chardon F (2012) Natural variation of *Arabidopsis* response to nitrogen availability. Journal of Experimental Botany 63: 91–105. 21914659 10.1093/jxb/err244

[8] Ferrier T, Matus JT, Jin J, Riechmann JL (2011) *Arabidopsis* paves the way: genomic and network analyses in crops. Current Opinion in Biotechnology 22: 260–270. 21167706 10.1016/j.copbio.2010.11.010

[9] Koornneef M, Meinke D (2010) The development of *Arabidopsis* as a model plant. The Plant Journal 61: 909–921. 20409266 10.1111/j.1365-313X.2009.04086.x

[10] Somerville C, Koornneef M (2002) A fortunate choice: the history of *Arabidopsis* as a model plant. Nat Rev Genet 3: 883–889. 12415318 10.1038/nrg927

[11] Hoffmann MH (2002) Biogeography of *Arabidopsis* thaliana (L.) Heynh. (Brassicaceae). Journal of Biogeography 29: 125–134.

[12] Kover PX, Valdar W, Trakalo J, Scarcelli N, Ehrenreich IM, et al (2009) A Multiparent Advanced Generation Inter-Cross to Fine-Map Quantitative Traits in *Arabidopsis thaliana* . PLoS Genet 5: e1000551. 19593375 10.1371/journal.pgen.1000551 PMC2700969

[13] Pérez-Pérez JM, Rubio-Díaz S, Dhondt S, Hernández-Romero D, Sánchez-Soriano J, et al (2011) Whole organ, venation and epidermal cell morphological variations are correlated in the leaves of *Arabidopsis* mutants. Plant, Cell & Environment 34: 2200–2211. 10.1111/j.1365-3040.2011.02415.x 21883289

[14] Arvidsson S, Pérez-Rodríguez P, Mueller-Roeber B (2011) A growth phenotyping pipeline for *Arabidopsis thaliana* integrating image analysis and rosette area modeling for robust quantification of genotype effects. New Phytologist 191: 895–907. 21569033 10.1111/j.1469-8137.2011.03756.x

[15] Granier C, Aguirrezabal L, Chenu K, Cookson SJ, Dauzat M, et al (2006) PHENOPSIS, an automated platform for reproducible phenotyping of plant responses to soil water deficit in *Arabidopsis thaliana* permitted the identification of an accession with low sensitivity to soil water deficit. New Phytologist 169: 623–635. 16411964 10.1111/j.1469-8137.2005.01609.x

[16] Walter A, Scharr H, Gilmer F, Zierer R, Nagel KA, et al (2007) Dynamics of seedling growth acclimation towards altered light conditions can be quantified via GROWSCREEN: a setup and procedure designed for rapid optical phenotyping of different plant species. New Phytologist 174: 447–455. 17388907 10.1111/j.1469-8137.2007.02002.x

[17] Bensmihen S, Hanna AI, Langlade NB, Micol JL, Bangham A, et al (2008) Mutational spaces for leaf shape and size. HFSP Journal 2: 110–120. 19404477 10.2976/1.2836738 PMC2645570

[18] Weight C, Parnham D, Waites R (2008) LeafAnalyser: a computational method for rapid and large-scale analyses of leaf shape variation. The Plant Journal 53: 578–586. 18028263 10.1111/j.1365-313X.2007.03330.x

[19] Gonzalez RC, Woods RE (2002) Digital Image Processing. Upper Saddle River, NJ, USA: Prentice Hall.

[20] Devlin PF, Halliday KJ, Harberd NP, Whitelam GC (1996) The rosette habit of *Arabidopsis thaliana* is dependent upon phytochrome action: novel phytochromes control internode elongation and flowering time. The Plant Journal 10: 1127–1134. 9011093 10.1046/j.1365-313x.1996.10061127.x

[21] Preuss D (2002) How to grow *Arabidopsis*. In: Weigel D, Glazebrook J, editors. In Arabidopsis, A Laboratory Manual: Cold Spring Harbour Laboratory Press, New York.

[22] Lemnatec (2010) LemnaLauncher Image analysis. LemnaLauncher and LemnaMiner Manual. Würselen, Germany: Lemnatec. \pp. 112–186.

[23] The MathWorks I (2012b) MATLAB and Statistics Toolbox Release. Natick, Massachusetts, United States.

[24] Pinheiro J, Bates D (2000) Mixed-Effects Models in S and S-PLUS: Springer: New York.

[25] R Development Core Team (2011) R: A Language and Environment for Statistical Computing. R Foundation for Statistical Computing.

[26] Hall M, Eibe F, Holmes G, Pfahringer B, Reutemann P, et al. (2009) The WEKA Data Mining Software: An Update. SIGKDD Explorations 11..

[27] Robnik-Šikonja M, Kononenko I (1997) An adaptation of Relief for attribute estimation in regression. Proceedings of the Fourteenth International Conference on Machine Learning 296–304.

[28] Clark RT, MacCurdy RB, Jung JK, Shaff JE, McCouch SR, et al (2011) Three-Dimensional Root Phenotyping with a Novel Imaging and Software Platform. Plant Physiology 156: 455–465. 21454799 10.1104/pp.110.169102 PMC3177249

[29] de Dorlodot S, Forster B, Pagès L, Price A, Tuberosa R, et al (2007) Root system architecture: opportunities and constraints for genetic improvement of crops. Trends in Plant Science 12: 474–481. 17822944 10.1016/j.tplants.2007.08.012

[30] Casler MD, Undersander DJ (2006) Selection for Establishment Capacity in Reed Canarygrass. Crop Science 46: 1277–1285.

[31] Yang Y, Costa A, Leonhardt N, Siegel R, Schroeder J (2008) Isolation of a strong *Arabidopsis* guard cell promoter and its potential as a research tool. Plant Methods 4: 6. 18284694 10.1186/1746-4811-4-6 PMC2323621

[32] Paproki A, Sirault X, Berry S, Furbank R, Fripp J (2012) A novel mesh processing based technique for 3D plant analysis. BMC Plant Biology 12: 63. 22553969 10.1186/1471-2229-12-63 PMC3464618

[33] Côté J-F, Fournier RA, Frazer GW, Niemann KO (2012) A fine-scale architectural model of trees to enhance LiDAR-derived measurements of forest canopy structure. Agricultural and Forest Meteorology 166–167: 72–85.

[34] Massonnet C, Vile D, Fabre J, Hannah MA, Caldana C, et al (2010) Probing the Reproducibility of Leaf Growth and Molecular Phenotypes: A Comparison of Three *Arabidopsis* Accessions Cultivated in Ten Laboratories. Plant Physiology 152: 2142–2157. 20200072 10.1104/pp.109.148338 PMC2850010

[35] Weight C, Parnham D, Waites R (2008) Techincal Advance: LeafAnalyser: a computational method for rapid and large-scale analyses of leaf shape variation. The Plant Journal 53: 578–586. 18028263 10.1111/j.1365-313X.2007.03330.x

[36] Schneider CA, Rasband WS, Eliceiri KW (2012) NIH Image to ImageJ: 25 years of image analysis. Nature Methods 9: 671–675. 22930834 10.1038/nmeth.2089 PMC5554542

[37] Octave community (2014) GNU Octave 3.8.

[38] Scilab Enterprises (2012) Scilab: Free and Open Source software for numerical computation.

[39] Sandve GK, Nekrutenko A, Taylor J, Hovig E (2013) Ten Simple Rules for Reproducible Computational Research. PLoS Comput Biol 9: e1003285. 24204232 10.1371/journal.pcbi.1003285 PMC3812051

