# Supplementary figures and images for: Objective Definition of Rosette Shape Variation Using a Combined Computer Vision and Data Mining Approach
Source: PLoS One. 2014 May 7;9(5):e96889. doi: 10.1371/journal.pone.0096889 (PMC4013065; doi:10.1371/journal.pone.0096889)

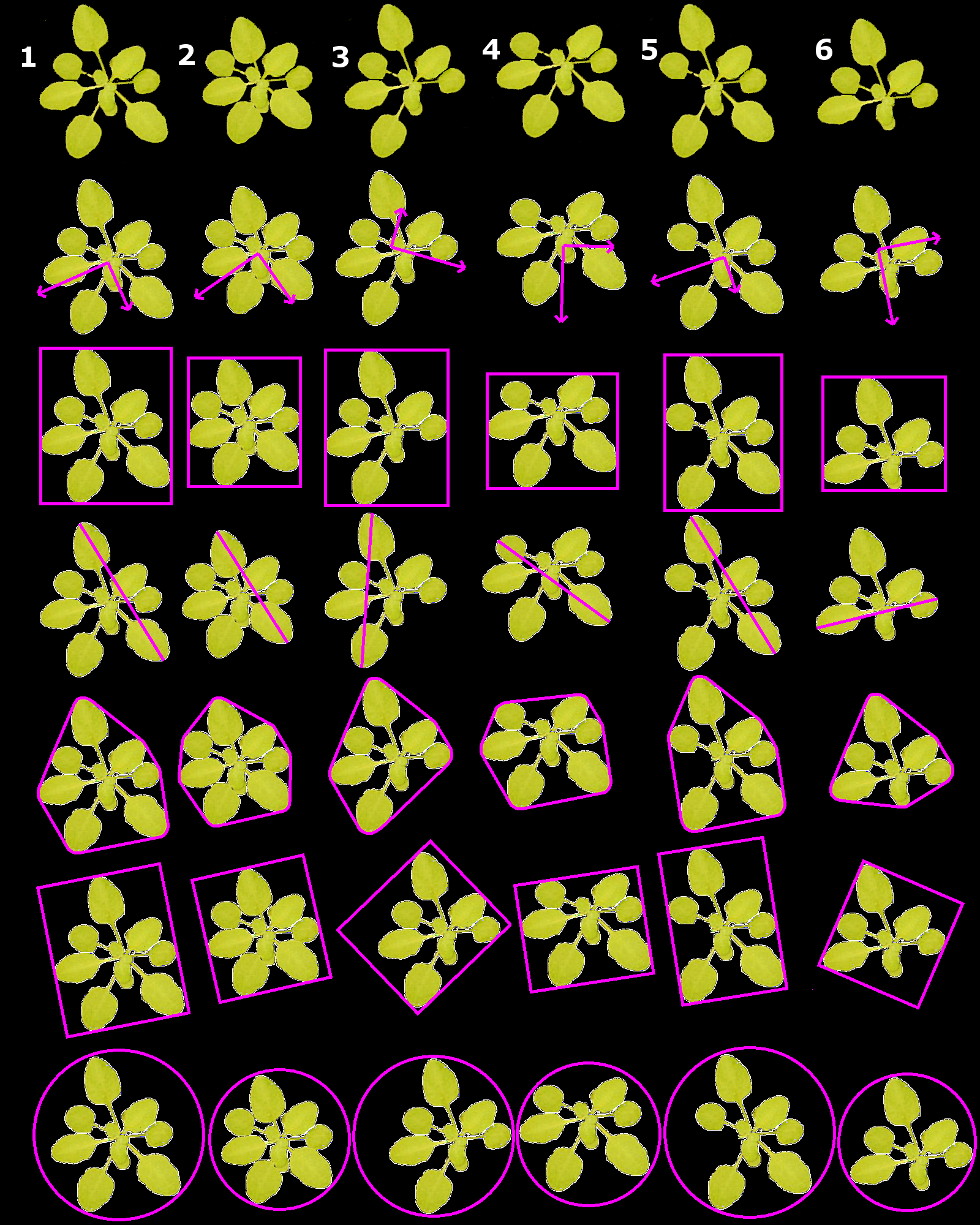

Supplement: Figure S1 — Illustration of variation in the shapes of Arabidopsis rosettes and of descriptors (as defined in Tables 1 and S1, with values given in table S2. Rows in order from top: Segmented image; Normsmallpax and Normlargepax; Vrectsizex and Vrectsizey; Maxdiam; convex hull from which Conhullarea and Conhullcirc are derived; Rectangle from which Minrectarea is determined and (bottom row) the circle from which Mincirclediam is determined. The rosettes in the image are artificial, they correspond to one Arabidopsis rosette manually modified to demonstrate the meaning of the descriptors. (TIF) [file pone.0096889.s001.tif]

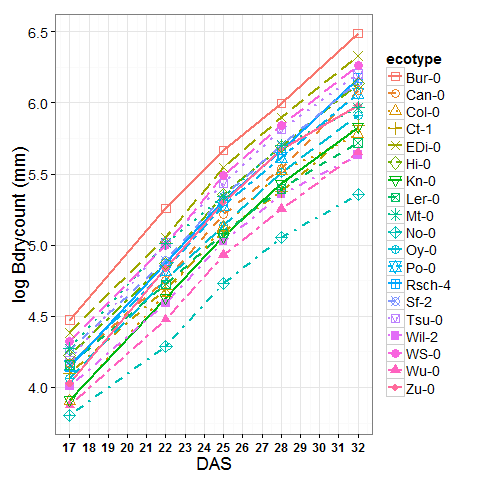

Supplement: Figure S2 — Time courses. Shape and size descriptors and area growth rate for the 19 Ecotypes. Values are averages per ecotype at each time point. (ZIP) [file pone.0096889.s002.zip › IntplotBdrycount.png]

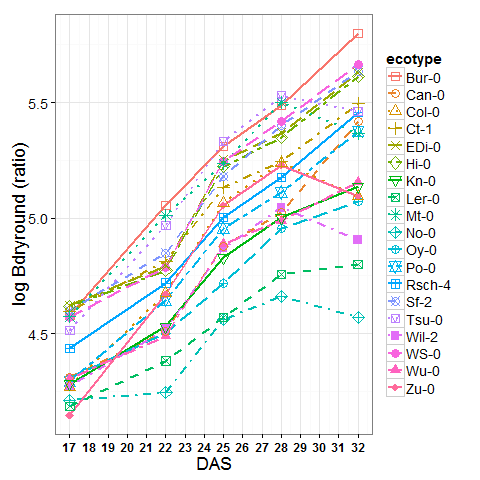

Supplement: Figure S2 — Time courses. Shape and size descriptors and area growth rate for the 19 Ecotypes. Values are averages per ecotype at each time point. (ZIP) [file pone.0096889.s002.zip › IntplotBdryround.png]

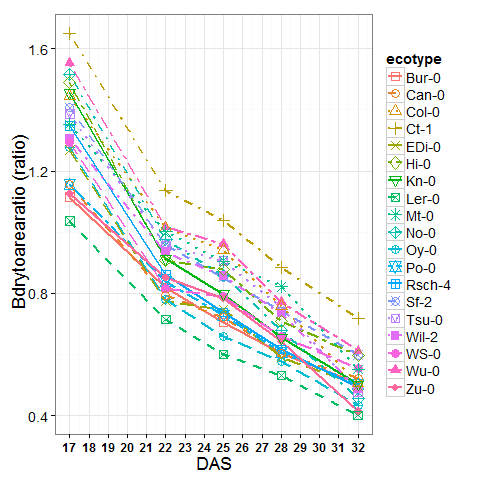

Supplement: Figure S2 — Time courses. Shape and size descriptors and area growth rate for the 19 Ecotypes. Values are averages per ecotype at each time point. (ZIP) [file pone.0096889.s002.zip › IntplotBdrytoarearatio.png]

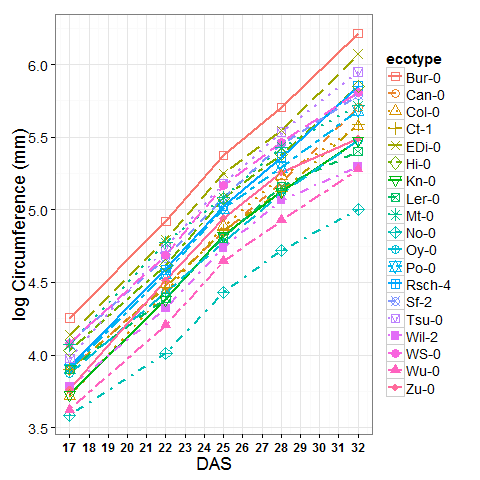

Supplement: Figure S2 — Time courses. Shape and size descriptors and area growth rate for the 19 Ecotypes. Values are averages per ecotype at each time point. (ZIP) [file pone.0096889.s002.zip › IntplotCircumference.png]

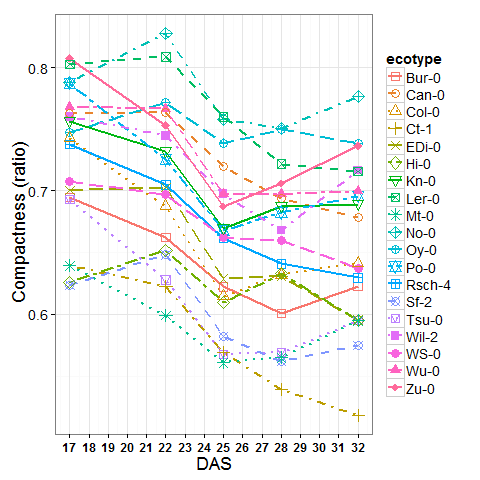

Supplement: Figure S2 — Time courses. Shape and size descriptors and area growth rate for the 19 Ecotypes. Values are averages per ecotype at each time point. (ZIP) [file pone.0096889.s002.zip › IntplotCompactness.png]

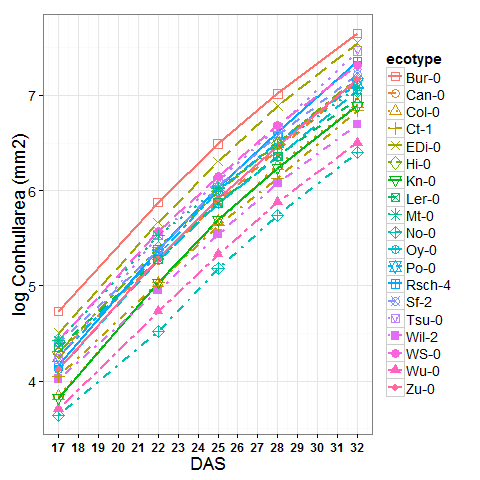

Supplement: Figure S2 — Time courses. Shape and size descriptors and area growth rate for the 19 Ecotypes. Values are averages per ecotype at each time point. (ZIP) [file pone.0096889.s002.zip › IntplotConhullarea.png]

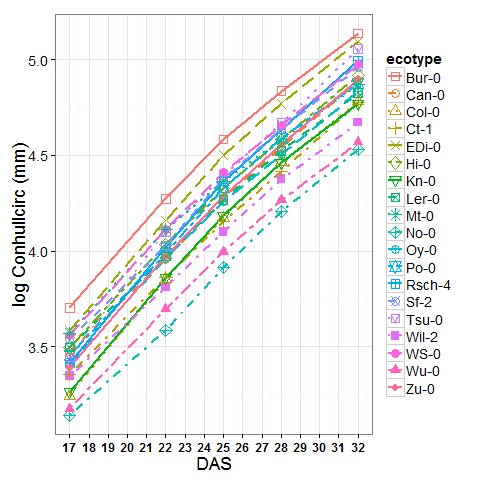

Supplement: Figure S2 — Time courses. Shape and size descriptors and area growth rate for the 19 Ecotypes. Values are averages per ecotype at each time point. (ZIP) [file pone.0096889.s002.zip › IntplotConhullcirc.png]

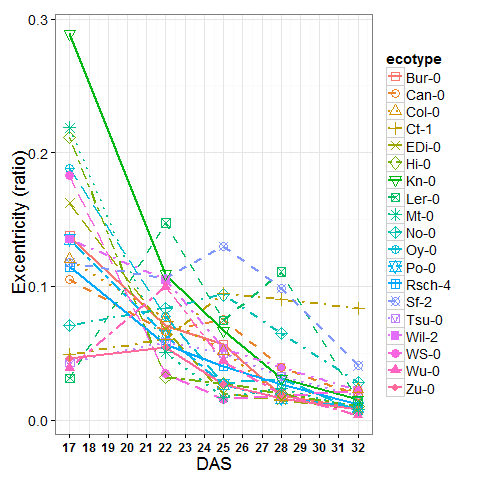

Supplement: Figure S2 — Time courses. Shape and size descriptors and area growth rate for the 19 Ecotypes. Values are averages per ecotype at each time point. (ZIP) [file pone.0096889.s002.zip › IntplotExcentricity.png]

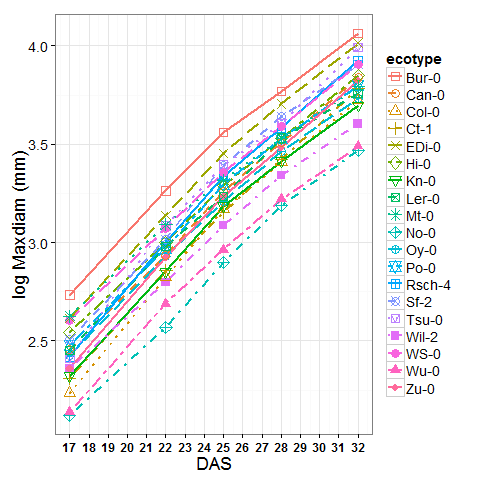

Supplement: Figure S2 — Time courses. Shape and size descriptors and area growth rate for the 19 Ecotypes. Values are averages per ecotype at each time point. (ZIP) [file pone.0096889.s002.zip › IntplotMaxdiam.png]

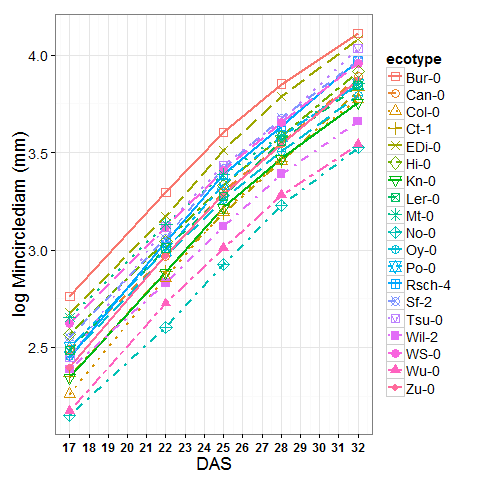

Supplement: Figure S2 — Time courses. Shape and size descriptors and area growth rate for the 19 Ecotypes. Values are averages per ecotype at each time point. (ZIP) [file pone.0096889.s002.zip › IntplotMincirclediam.png]

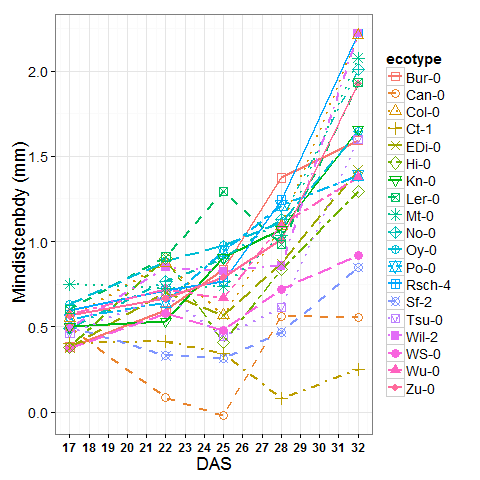

Supplement: Figure S2 — Time courses. Shape and size descriptors and area growth rate for the 19 Ecotypes. Values are averages per ecotype at each time point. (ZIP) [file pone.0096889.s002.zip › IntplotMindistcenbdy.png]

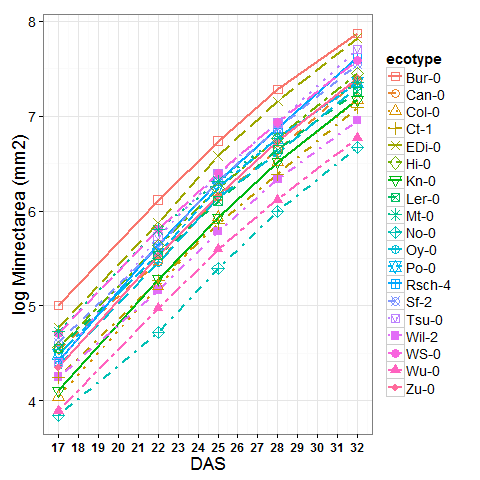

Supplement: Figure S2 — Time courses. Shape and size descriptors and area growth rate for the 19 Ecotypes. Values are averages per ecotype at each time point. (ZIP) [file pone.0096889.s002.zip › IntplotMinrectarea.png]

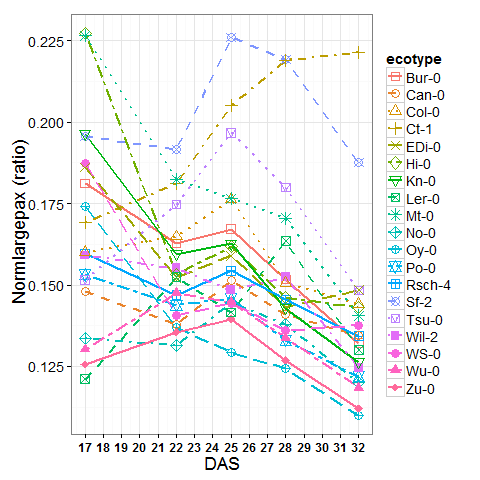

Supplement: Figure S2 — Time courses. Shape and size descriptors and area growth rate for the 19 Ecotypes. Values are averages per ecotype at each time point. (ZIP) [file pone.0096889.s002.zip › IntplotNormlargepax.png]

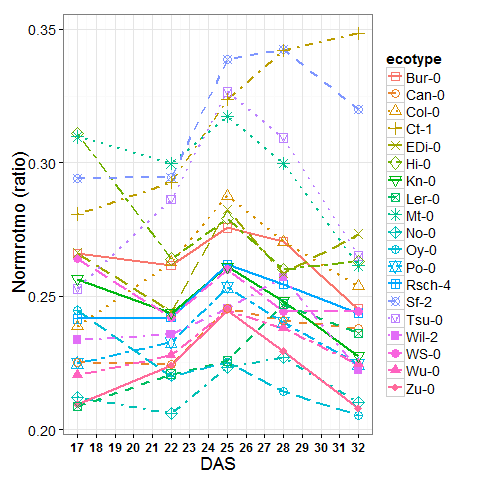

Supplement: Figure S2 — Time courses. Shape and size descriptors and area growth rate for the 19 Ecotypes. Values are averages per ecotype at each time point. (ZIP) [file pone.0096889.s002.zip › IntplotNormrotmo.png]

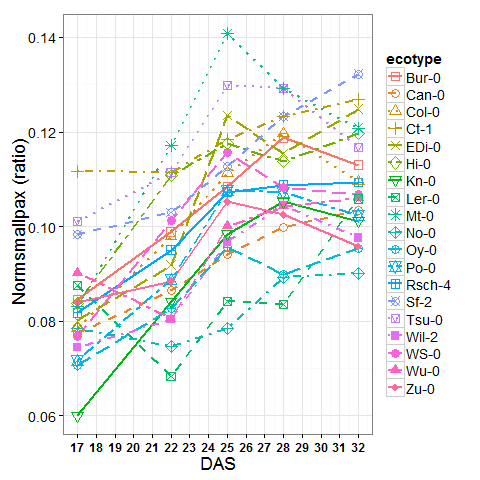

Supplement: Figure S2 — Time courses. Shape and size descriptors and area growth rate for the 19 Ecotypes. Values are averages per ecotype at each time point. (ZIP) [file pone.0096889.s002.zip › IntplotNormsmallpax.png]

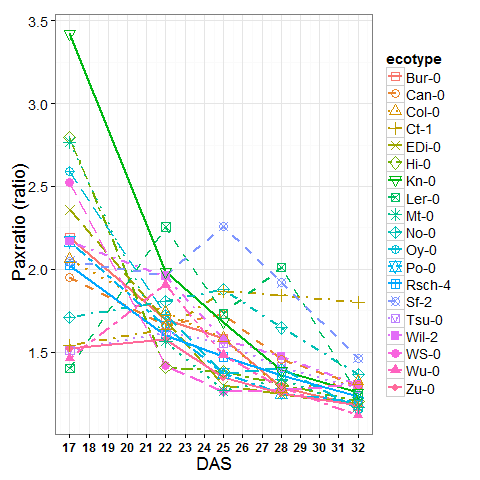

Supplement: Figure S2 — Time courses. Shape and size descriptors and area growth rate for the 19 Ecotypes. Values are averages per ecotype at each time point. (ZIP) [file pone.0096889.s002.zip › IntplotPaxratio.png]

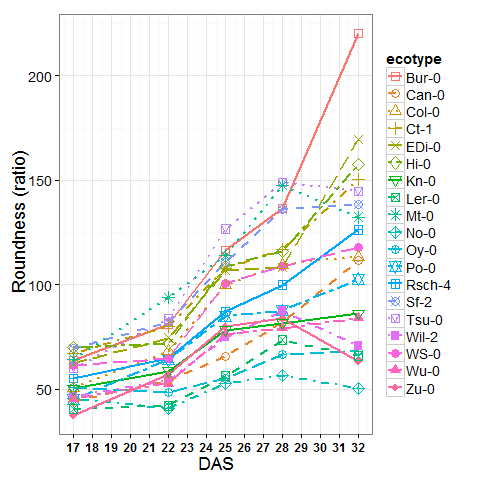

Supplement: Figure S2 — Time courses. Shape and size descriptors and area growth rate for the 19 Ecotypes. Values are averages per ecotype at each time point. (ZIP) [file pone.0096889.s002.zip › IntplotRoundness.png]

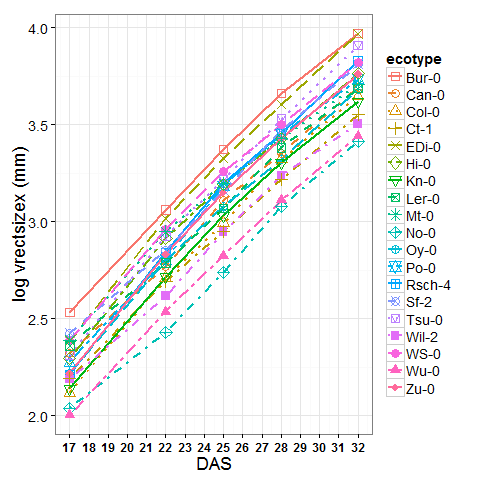

Supplement: Figure S2 — Time courses. Shape and size descriptors and area growth rate for the 19 Ecotypes. Values are averages per ecotype at each time point. (ZIP) [file pone.0096889.s002.zip › Intplotvrectsizex.png]

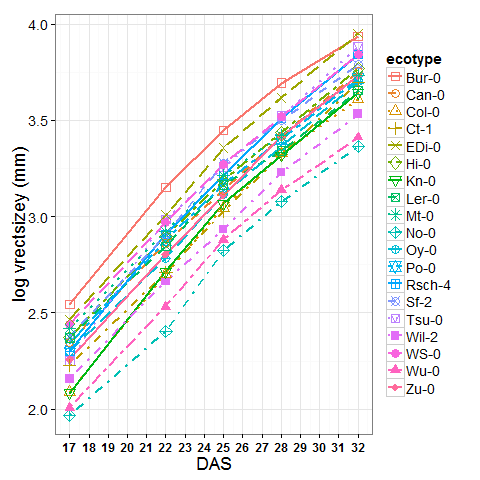

Supplement: Figure S2 — Time courses. Shape and size descriptors and area growth rate for the 19 Ecotypes. Values are averages per ecotype at each time point. (ZIP) [file pone.0096889.s002.zip › Intplotvrectsizey.png]

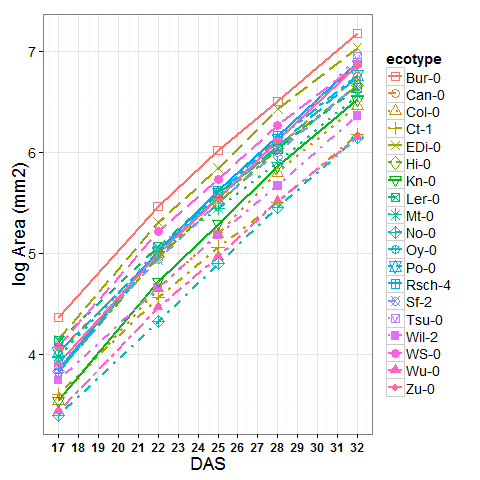

Supplement: Figure S2 — Time courses. Shape and size descriptors and area growth rate for the 19 Ecotypes. Values are averages per ecotype at each time point. (ZIP) [file pone.0096889.s002.zip › IntplotArea.png]

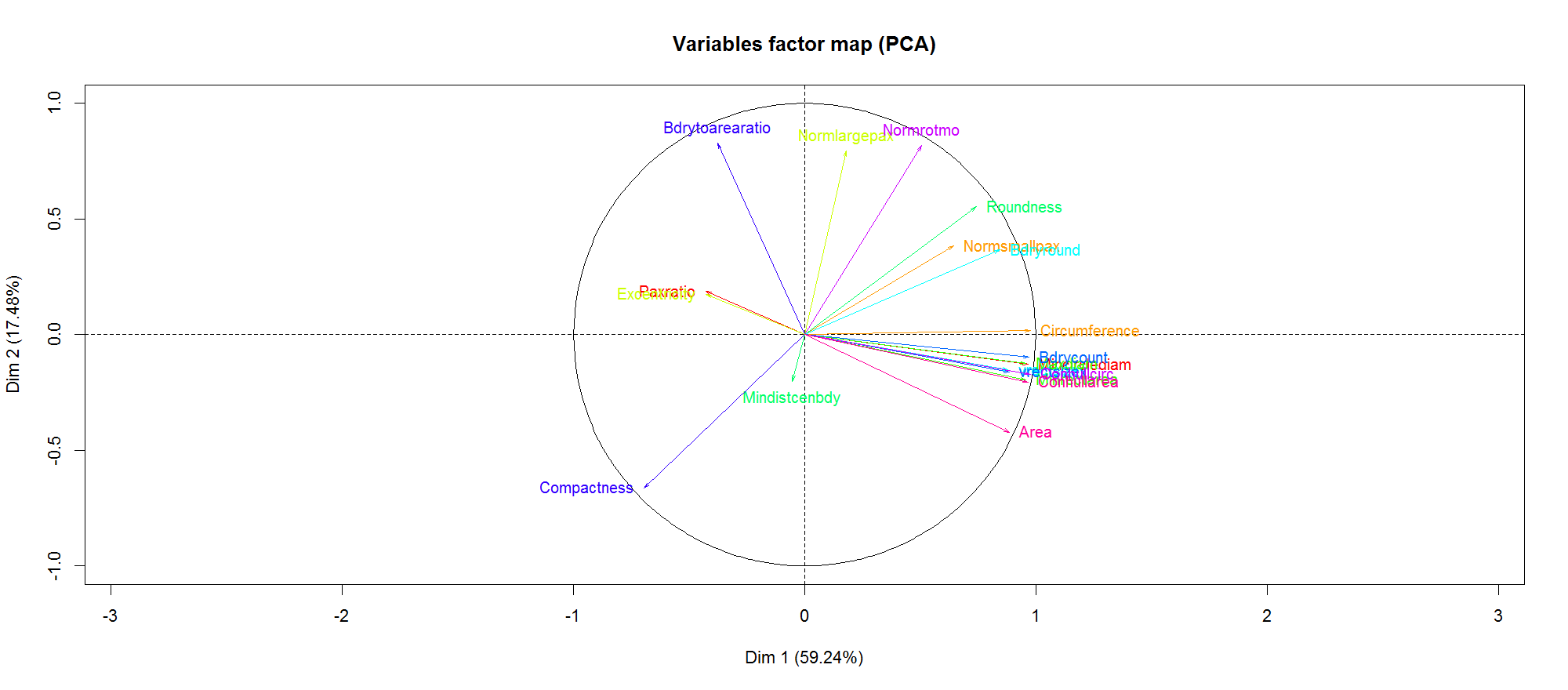

Supplement: Figure S3 — PCA at successive time points. (A) 22 DAS, (B) 25 DAS, (C) 25 DAS and (D) across all-time series. (ZIP) [file pone.0096889.s003.zip › PCAalldescriptors_time22.png]

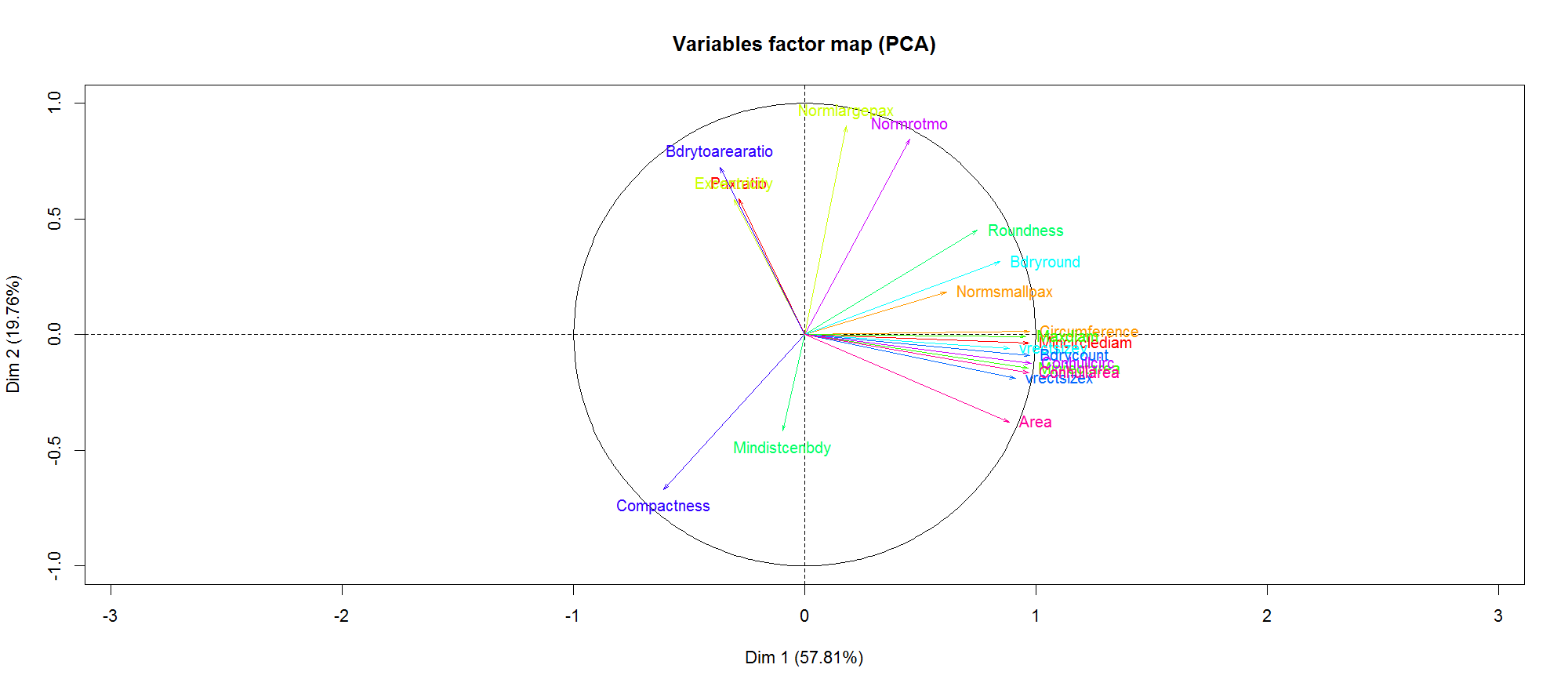

Supplement: Figure S3 — PCA at successive time points. (A) 22 DAS, (B) 25 DAS, (C) 25 DAS and (D) across all-time series. (ZIP) [file pone.0096889.s003.zip › PCAalldescriptors_time25.png]

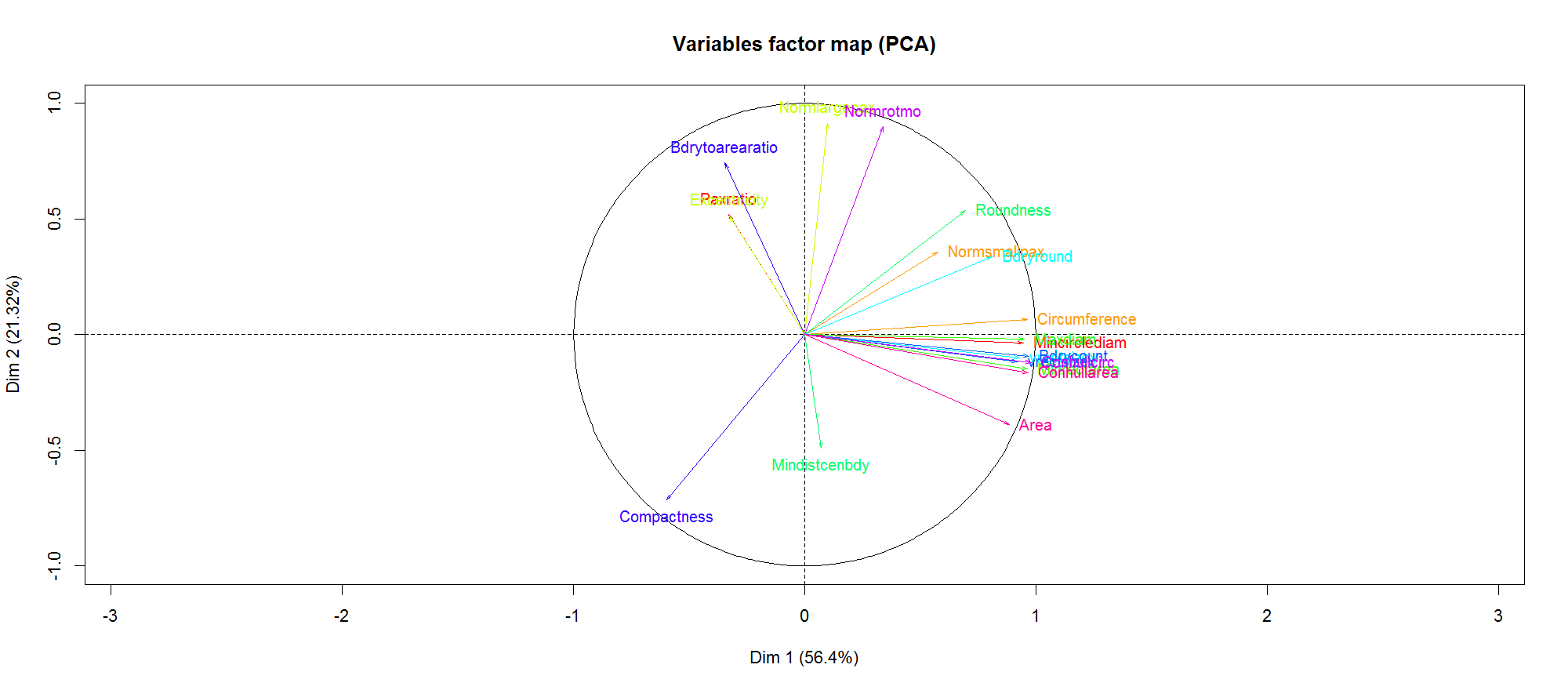

Supplement: Figure S3 — PCA at successive time points. (A) 22 DAS, (B) 25 DAS, (C) 25 DAS and (D) across all-time series. (ZIP) [file pone.0096889.s003.zip › PCAalldescriptors_time28.png]

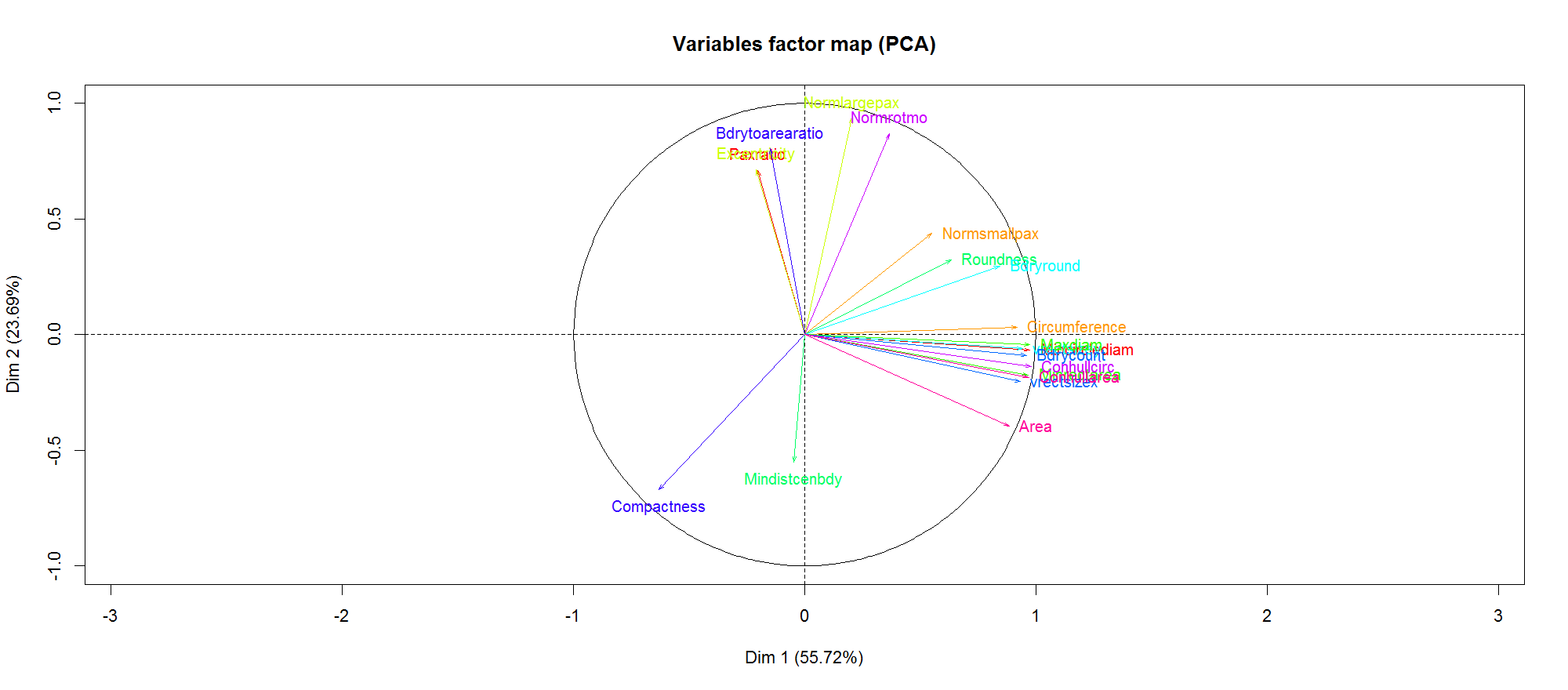

Supplement: Figure S3 — PCA at successive time points. (A) 22 DAS, (B) 25 DAS, (C) 25 DAS and (D) across all-time series. (ZIP) [file pone.0096889.s003.zip › PCAalldescriptors_time32.png]

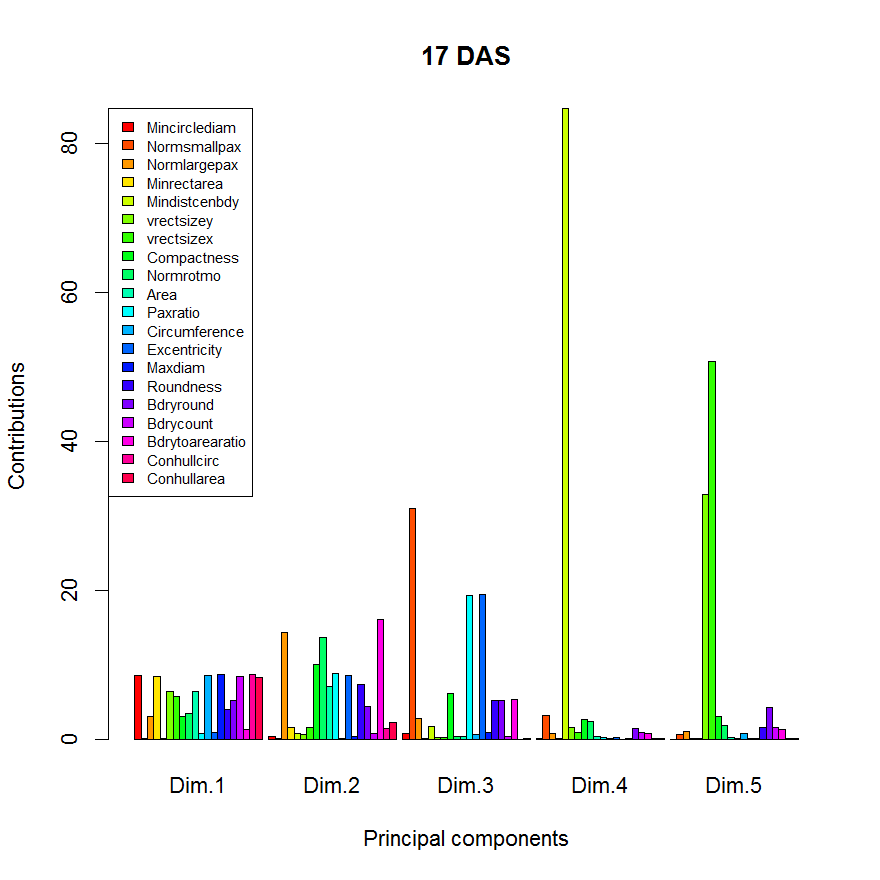

Supplement: Figure S4 — Relative contributions of all variables to principal components 1 to 5 for analysed separately 22, 25 28 and 32 DAS. (For 17 DAS see Figure 6). (ZIP) [file pone.0096889.s004.zip › ContribPCA_time17.png]

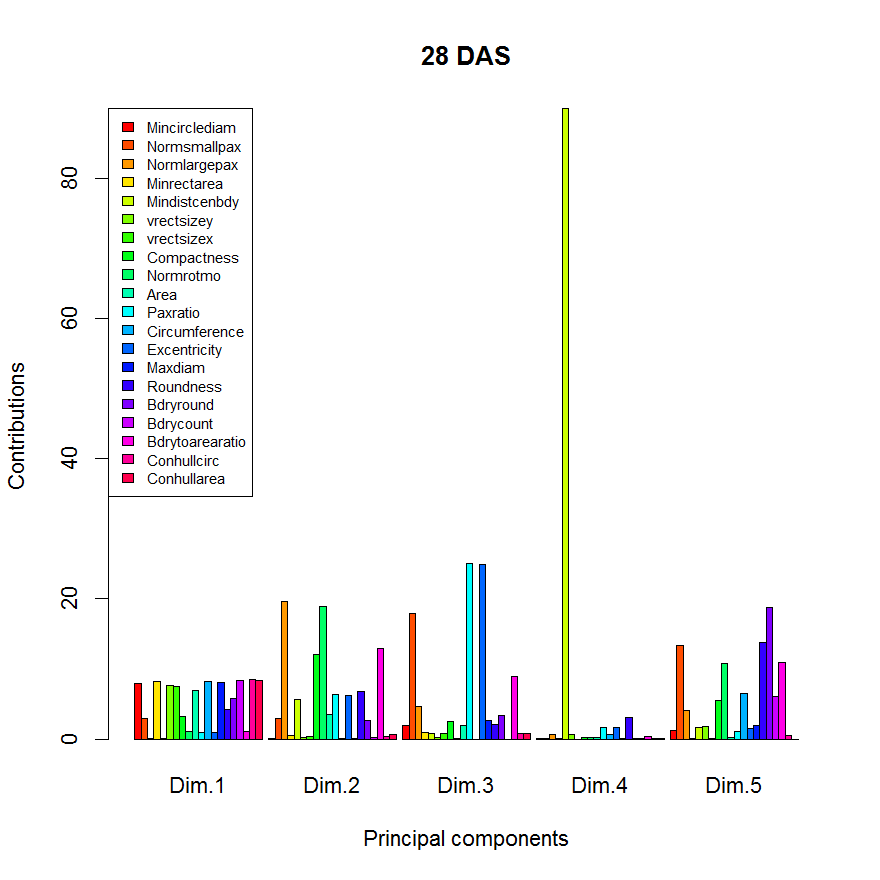

Supplement: Figure S4 — Relative contributions of all variables to principal components 1 to 5 for analysed separately 22, 25 28 and 32 DAS. (For 17 DAS see Figure 6). (ZIP) [file pone.0096889.s004.zip › ContribPCA_time28.png]

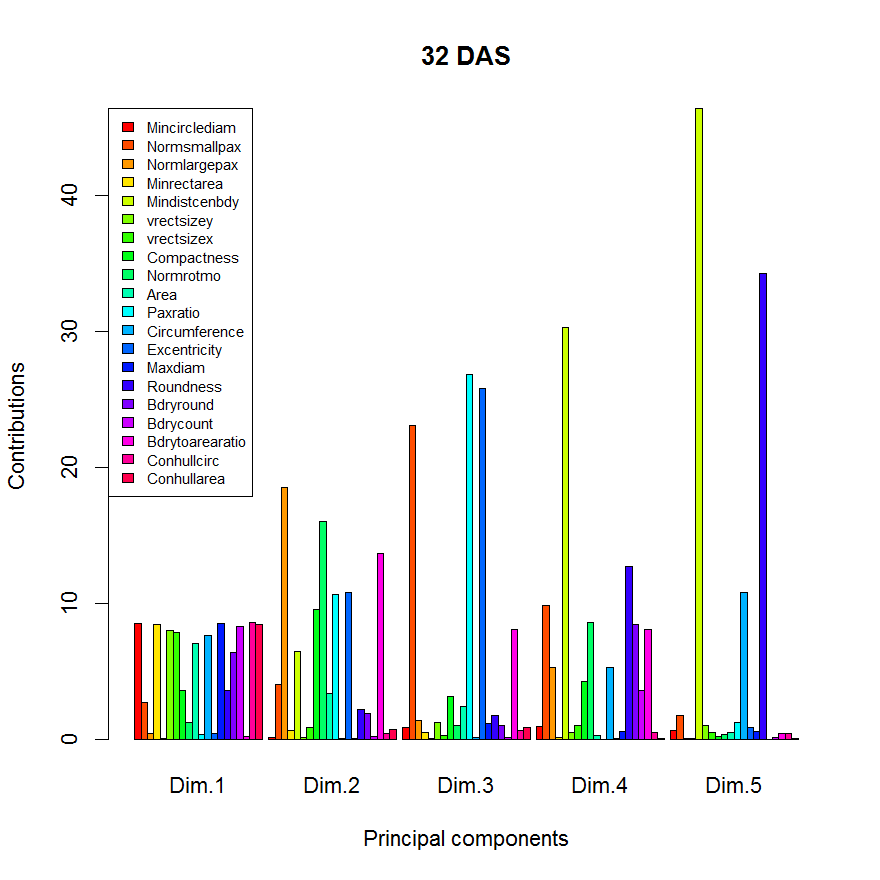

Supplement: Figure S4 — Relative contributions of all variables to principal components 1 to 5 for analysed separately 22, 25 28 and 32 DAS. (For 17 DAS see Figure 6). (ZIP) [file pone.0096889.s004.zip › ContribPCA_time32.png]

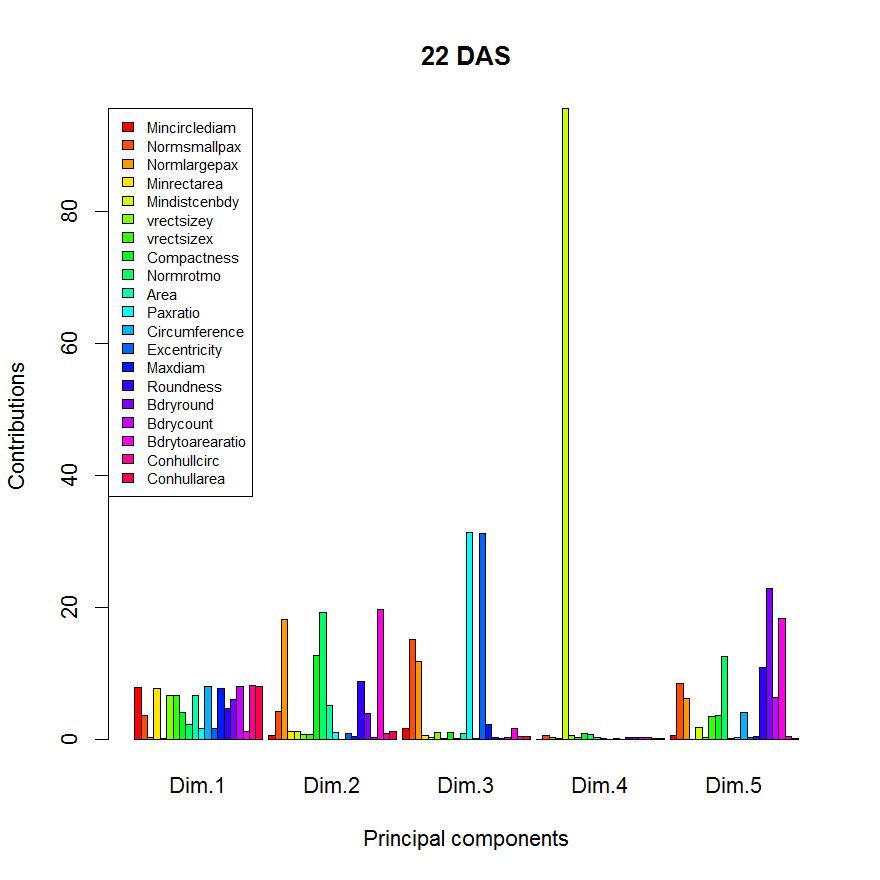

Supplement: Figure S4 — Relative contributions of all variables to principal components 1 to 5 for analysed separately 22, 25 28 and 32 DAS. (For 17 DAS see Figure 6). (ZIP) [file pone.0096889.s004.zip › ContribPCA_time22.png]

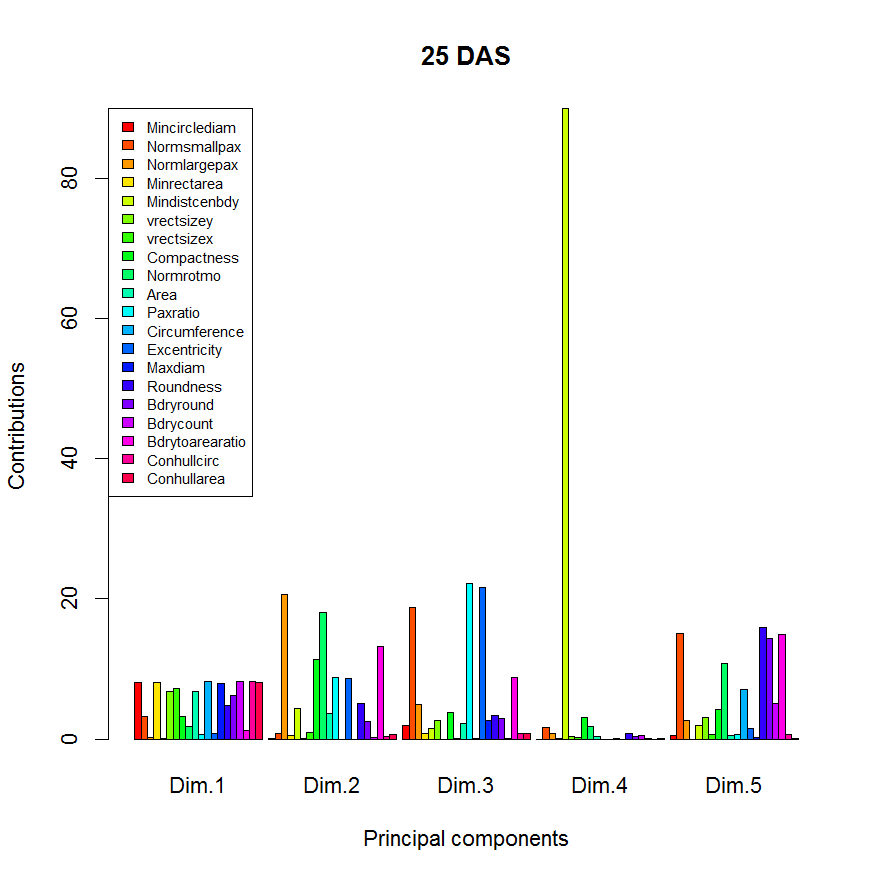

Supplement: Figure S4 — Relative contributions of all variables to principal components 1 to 5 for analysed separately 22, 25 28 and 32 DAS. (For 17 DAS see Figure 6). (ZIP) [file pone.0096889.s004.zip › ContribPCA_time25.png]

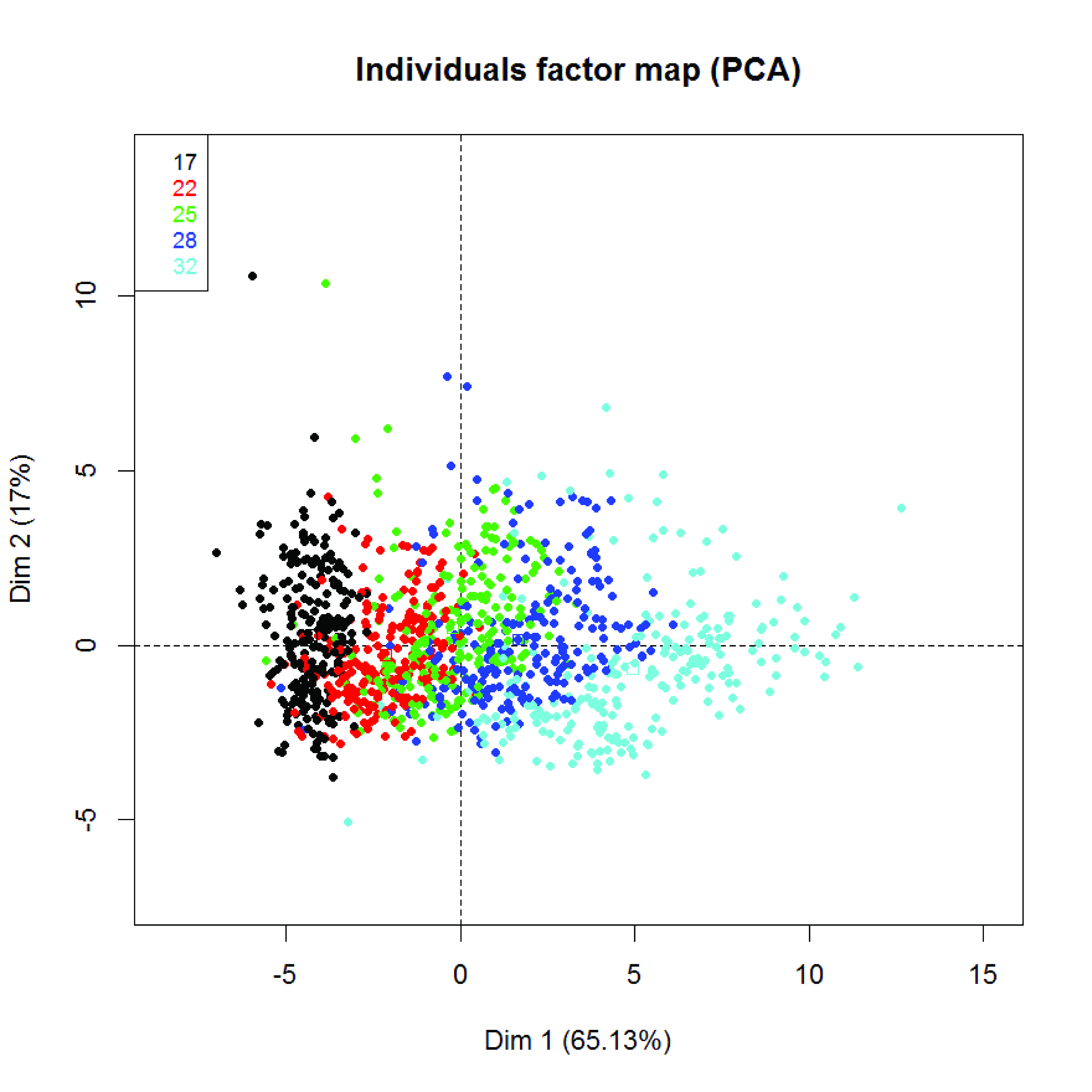

Supplement: Figure S5 — Principal components 1 and 2 from PCA combined over all ecotypes and times with points labelled by time. (TIF) [file pone.0096889.s005.tif]

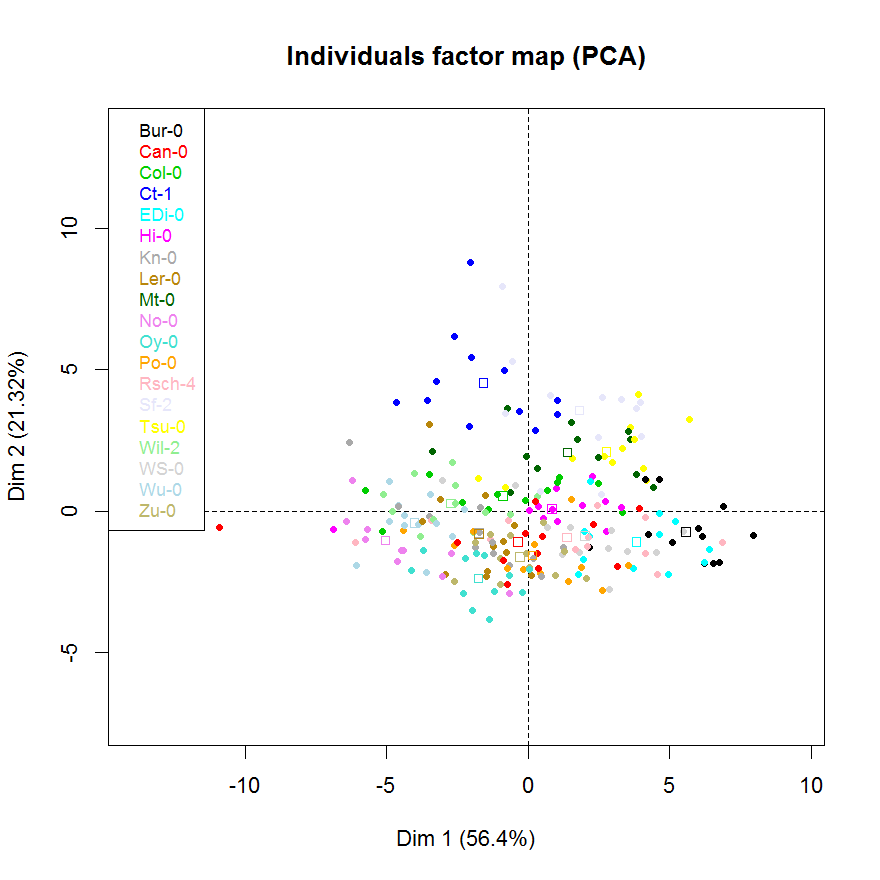

Supplement: Figure S6 — PCA For each time and grouped by ecotype. (A) 22 DAS, (B) 25 DAS, (C) 28 DAS and (D) 32 DAS. Small squares around the ecotypes show significant differences (P<0.05) between ecotypes. The smaller the square the more significant the difference. (ZIP) [file pone.0096889.s006.zip › PCA_by_ecotypetime_28.png]

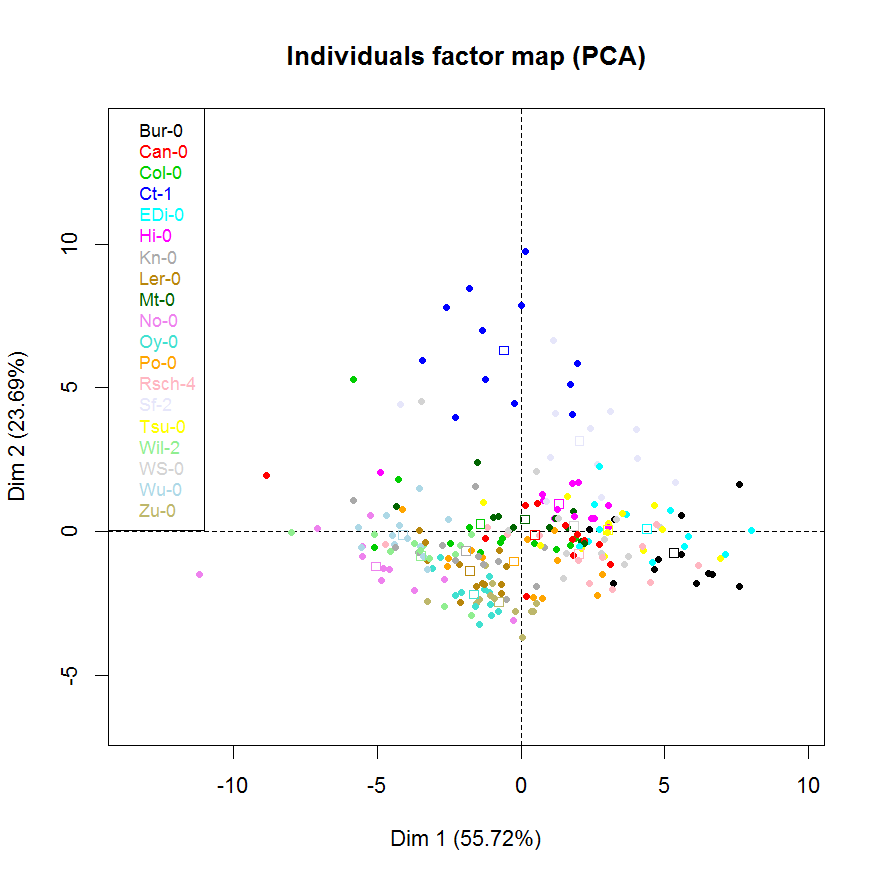

Supplement: Figure S6 — PCA For each time and grouped by ecotype. (A) 22 DAS, (B) 25 DAS, (C) 28 DAS and (D) 32 DAS. Small squares around the ecotypes show significant differences (P<0.05) between ecotypes. The smaller the square the more significant the difference. (ZIP) [file pone.0096889.s006.zip › PCA_by_ecotypetime_32.png]

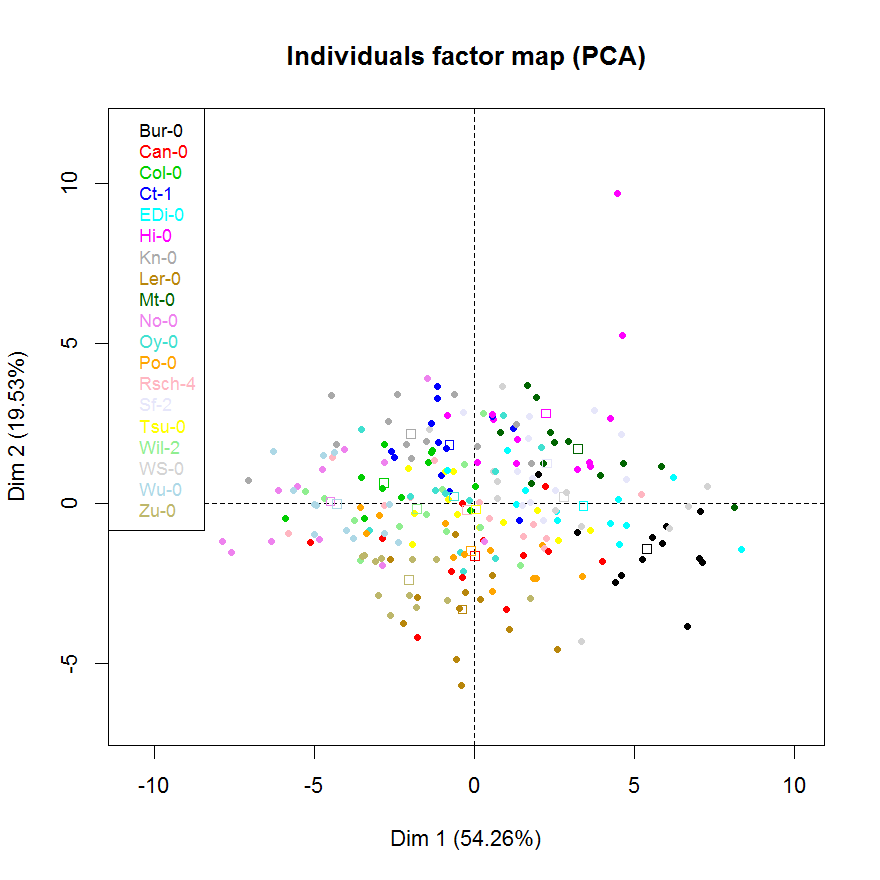

Supplement: Figure S6 — PCA For each time and grouped by ecotype. (A) 22 DAS, (B) 25 DAS, (C) 28 DAS and (D) 32 DAS. Small squares around the ecotypes show significant differences (P<0.05) between ecotypes. The smaller the square the more significant the difference. (ZIP) [file pone.0096889.s006.zip › PCA_by_ecotypetime_17.png]

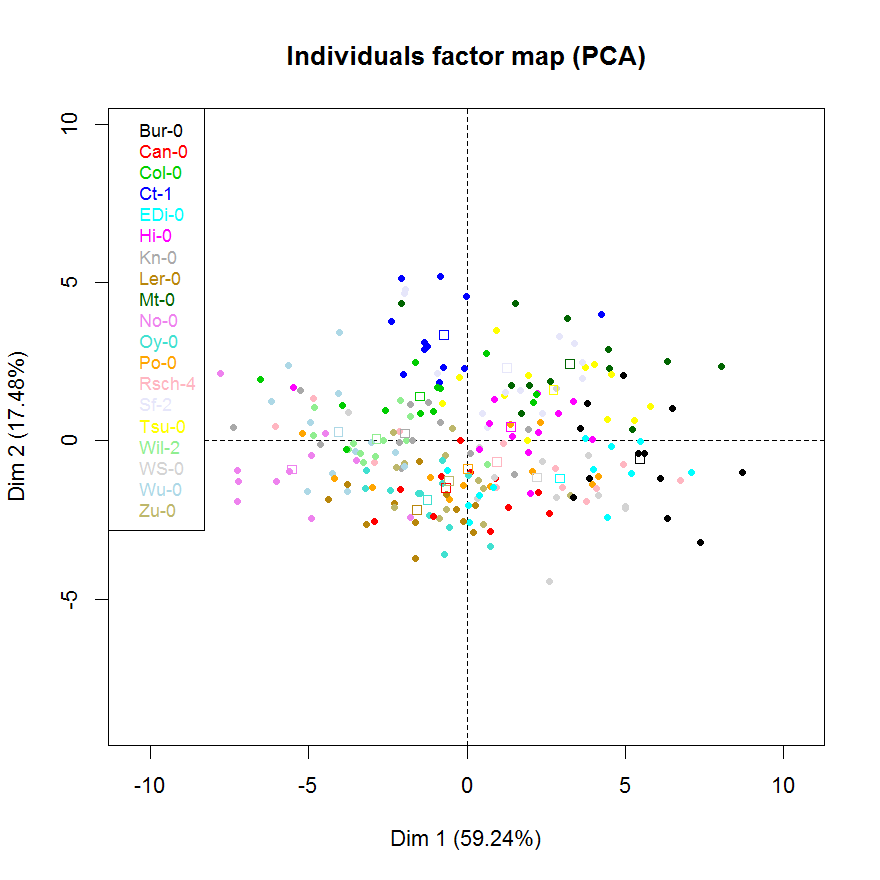

Supplement: Figure S6 — PCA For each time and grouped by ecotype. (A) 22 DAS, (B) 25 DAS, (C) 28 DAS and (D) 32 DAS. Small squares around the ecotypes show significant differences (P<0.05) between ecotypes. The smaller the square the more significant the difference. (ZIP) [file pone.0096889.s006.zip › PCA_by_ecotypetime_22.png]

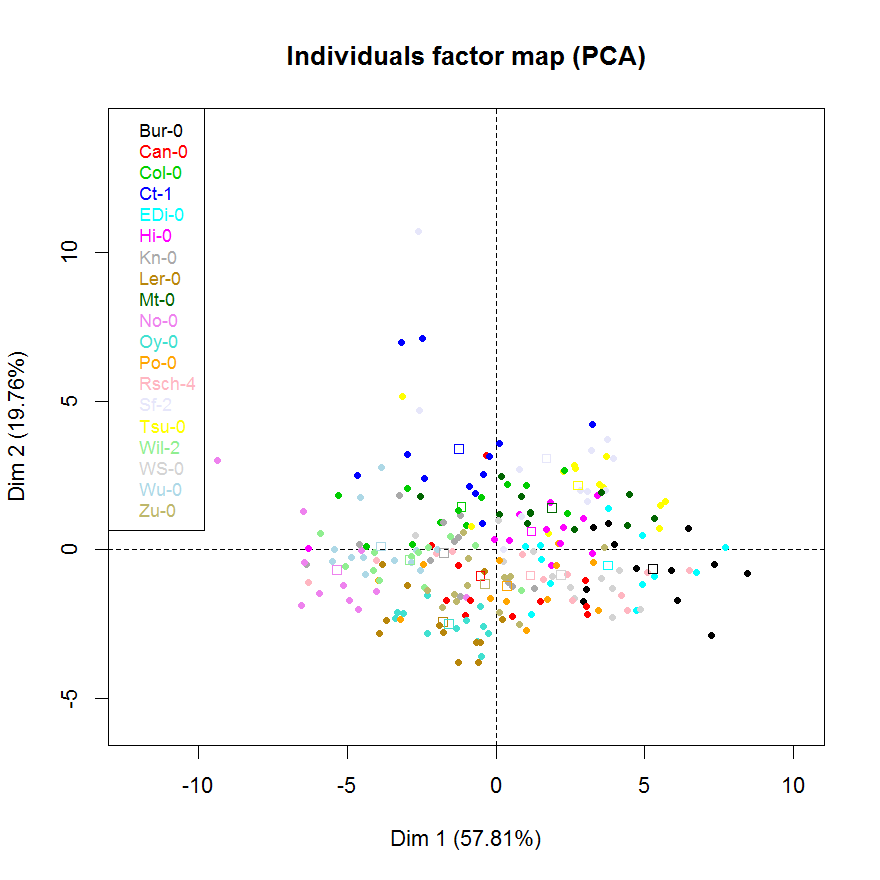

Supplement: Figure S6 — PCA For each time and grouped by ecotype. (A) 22 DAS, (B) 25 DAS, (C) 28 DAS and (D) 32 DAS. Small squares around the ecotypes show significant differences (P<0.05) between ecotypes. The smaller the square the more significant the difference. (ZIP) [file pone.0096889.s006.zip › PCA_by_ecotypetime_25.png]

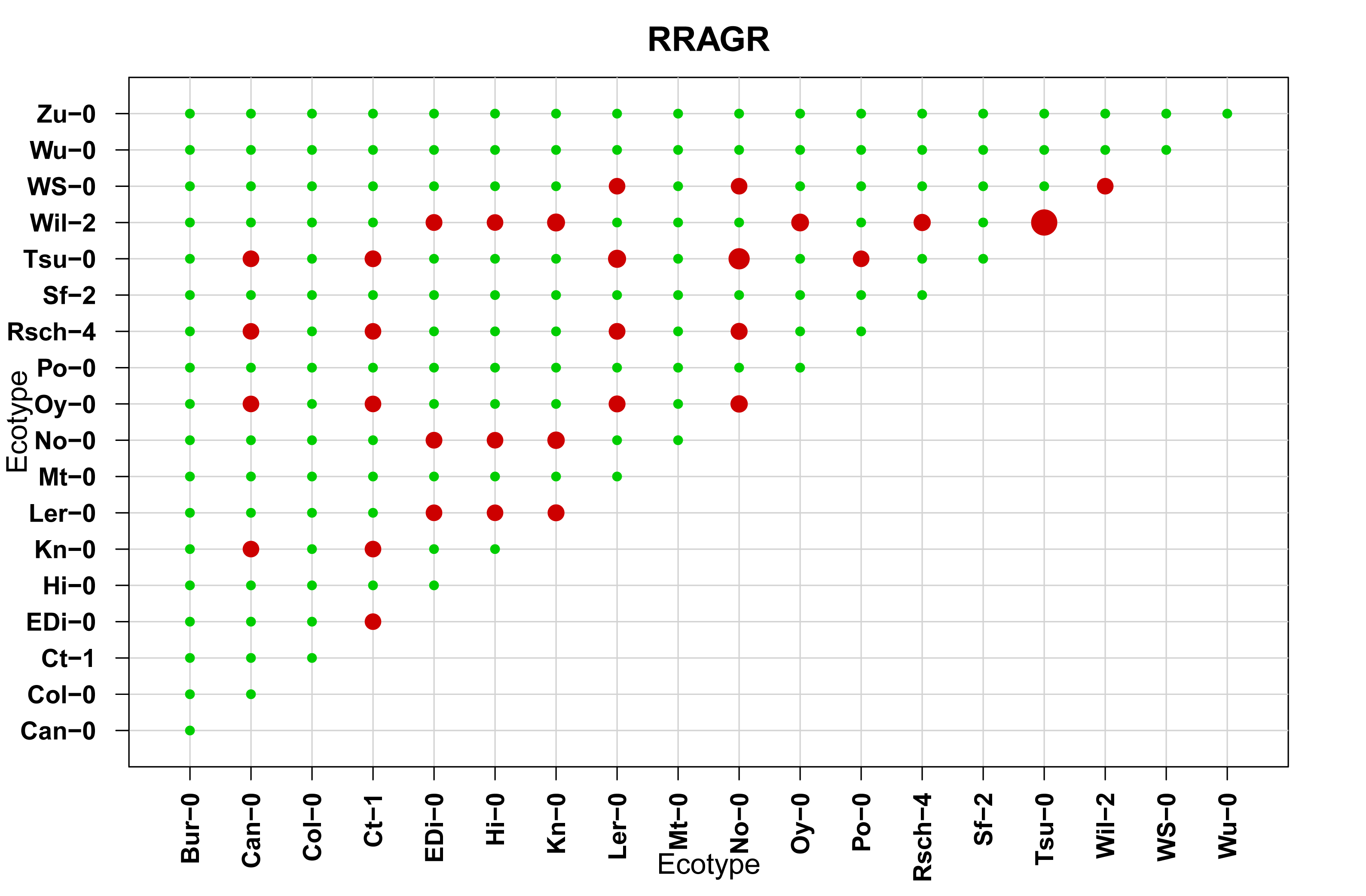

Supplement: Figure S7 — Multiple comparison of RRAGR. Scatter plots showing significant (P>0.05•, P<0.05 •, P<0.01•, Post-hoc Tukey test between ecotypes). (TIF) [file pone.0096889.s007.tif]

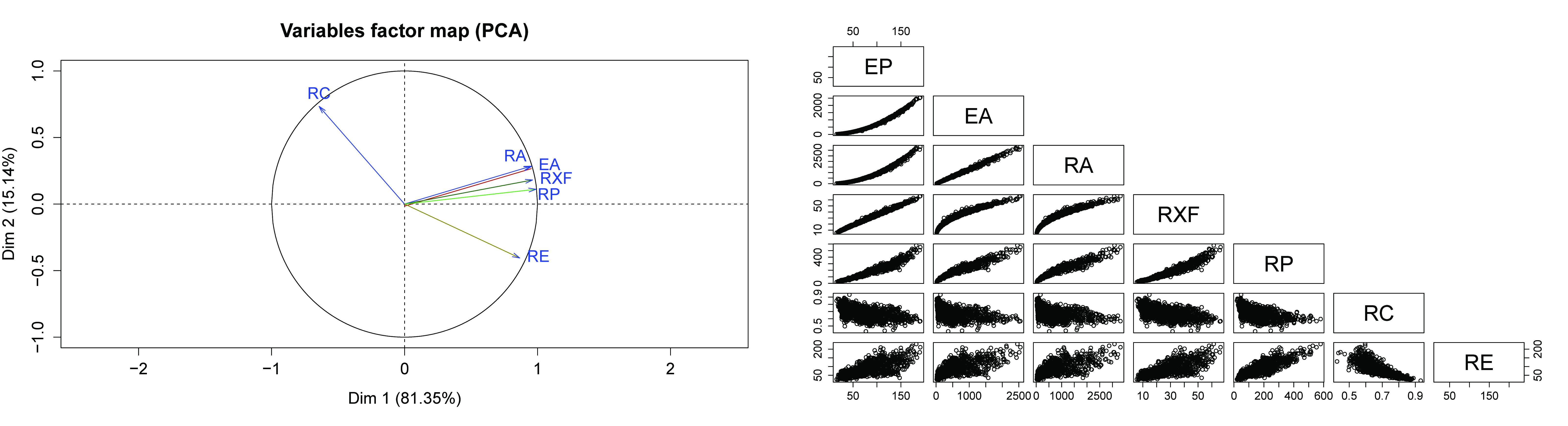

Supplement: Figure S8 — Results from analysis of descriptors here also used in [13] . (A) PCA and (B) Scatter plot showing relations between seven descriptors. (TIF) [file pone.0096889.s008.tif]
