# Supplementary material for: Objective Definition of Rosette Shape Variation Using a Combined Computer Vision and Data Mining Approach
Source: PLoS One. 2014 May 7;9(5):e96889. doi: 10.1371/journal.pone.0096889 (PMC4013065; doi:10.1371/journal.pone.0096889)
Supplement: Table S1 — Equivalence of names provided by LemnaTec software. (DOCX) [file pone.0096889.s009.docx]

# Table S1. Equivalence of names provided by LemnaTec software

| Id | Name | Name in LemnaTec software |
| --- | --- | --- |
| 1 | Mincirclediam | Min.Enclosing.Circle.Diameter |
|  | Normsmallpax | X2nd.Moment.Principle.Axis.Small.Norm |
|  | Normlargepax | X2nd.Moment.Principle.Axis.Large.Norm |
| 4 | Minrectarea | Min.Area.Rectangle.Area |
| 5 | Mindistcenbdy | Centre.Of.Mass.To.Boundary.Distance |
| 6 | vrectsizey | Object.Extent.Y |
| 7 | vrectsizex | Object.Extent.X |
| 8 | Compactness | Compactness |
| 9 | Normrotmo | Normalised.Z.Rotation.2nd.Moment |
| 0 | Area | Area |
|  | Paxratio | X2nd.Moments.Principal.Axis.Ratio |
| 2 | Circumference | Circumference |
| 3 | Excentricity | Excentricity |
| 4 | Maxdiam | Caliper.Length |
| 5 | Roundness | Roundness |
| 6 | Bdryround | Boundary.Point.Roundness |
| 7 | Bdrycount | Boundary.Point.Count |
| 8 | Bdrytoarearatio | Boundary.Points.To.Area.Ratio |
| 9 | Conhullcirc | Convex.Hull.Circumference |
| 0 | Conhullarea | Convex.Hull.Area |
