# Supplementary material for: Objective Definition of Rosette Shape Variation Using a Combined Computer Vision and Data Mining Approach
Source: PLoS One. 2014 May 7;9(5):e96889. doi: 10.1371/journal.pone.0096889 (PMC4013065; doi:10.1371/journal.pone.0096889)
Supplement: Table S2 — Values of features extracted from rosettes illustrated in Figure S1 used to show variation in rosette descriptors with id of descriptor as in Table 1 . (DOCX) [file pone.0096889.s010.docx]

Table S2. Values of features extracted from rosettes and for explanatory purpose. Image ids correspond to those in Figure S1, values correspond to those in Table 1 and Table S1.

| **Image**  **id** | **Shape descriptors extracted from segmented *Arabidopsis* rosettes** | | | | | | | | | | | | | | | | | | | |
| --- | --- | --- | --- | --- | --- | --- | --- | --- | --- | --- | --- | --- | --- | --- | --- | --- | --- | --- | --- | --- |
|  | **1** | **2** | **3** | **4** | **5** | **6** | **7** | **8** | **9** | **10** | **11** | **12** | **13** | **14** | **15** | **16** | **17** | **18** | **19** | **20** |
|  |  |  |  |  |  |  |  |  |  |  |  |  |  |  |  |  |  |  |  |  |
| 1 | 277.4 | 0.106 | 0.157 | 50233 | 0.414 | 254.0 | 213.0 | 0.646 | 0.264 | 23578 | 1.482 | 1635 | 0.038 | 263.1 | 113.4 | 191.5 | 2125 | 0.090 | 727.5 | 36526 |
| 2 | 228.2 | 0.085 | 0.109 | 37965 | 3.584 | 210.0 | 183.0 | 0.781 | 0.194 | 22168 | 1.272 | 908.3 | 0.014 | 215.8 | 37.22 | 128.2 | 1686 | 0.076 | 621.7 | 28392 |
| 3 | 261.2 | 0.095 | 0.183 | 39660 | 1.102 | 254.0 | 200.0 | 0.646 | 0.278 | 19452 | 1.916 | 1350 | 0.099 | 253.6 | 93.73 | 163.1 | 1781 | 0.092 | 670.8 | 30102 |
| 4 | 233.3 | 0.104 | 0.159 | 35549 | 13.20 | 187.0 | 213.0 | 0.638 | 0.262 | 19554 | 1.525 | 1347 | 0.043 | 226.2 | 92.74 | 162.9 | 1785 | 0.091 | 651.5 | 30647 |
| 5 | 277.4 | 0.101 | 0.207 | 42911 | 8.915 | 254.0 | 191.0 | 0.598 | 0.308 | 20454 | 2.055 | 1524 | 0.119 | 263.1 | 113.6 | 172.2 | 1877 | 0.092 | 713.4 | 34203 |
| 6 | 222.9 | 0.119 | 0.145 | 32595 | -1.482 | 185.0 | 200.0 | 0.672 | 0.264 | 15857 | 1.222 | 1095 | 0.010 | 202.8 | 75.68 | 137.6 | 1477 | 0.093 | 593.6 | 23600 |
|  |  |  |  |  |  |  |  |  |  |  |  |  |  |  |  |  |  |  |  |  |
