# Supplementary material for: Objective Definition of Rosette Shape Variation Using a Combined Computer Vision and Data Mining Approach
Source: PLoS One. 2014 May 7;9(5):e96889. doi: 10.1371/journal.pone.0096889 (PMC4013065; doi:10.1371/journal.pone.0096889)
Supplement: Table S3 — Comparison of experiments between Perez-Perez and Camargo studies. (DOCX) [file pone.0096889.s011.docx]

Table S3. Comparison of experiments between Perez-Perez and Camargo studies.

| Description | Perez-Perez, et al | Camargo, et al |
| --- | --- | --- |
| *Arabidopsis* materials | 3 Parents, 108 Mutants | 19 Ecotypes |
| Growth media | Agar | Compost |
| Container | Petri dishes, 20 plants per 150 mm dish | 1 plant per pot 47 x 47 x51 mm |
| Descriptors extracted | 10 | 20 |
| Plant size | Young rosettes | Maturing vegetative rosettes |
| Plant age, at measurement, days after stratification | 21 | 17-32 |
| Temperature °C | 20 | 23 day 20 night |
| PPFD µmol m^-2^ s^-1^ | 78 | 110 |
| Day length, h | 24 | 8 |
