# Supplementary material for: Objective Definition of Rosette Shape Variation Using a Combined Computer Vision and Data Mining Approach
Source: PLoS One. 2014 May 7;9(5):e96889. doi: 10.1371/journal.pone.0096889 (PMC4013065; doi:10.1371/journal.pone.0096889)
Supplement: Table S4 — Correspondence of shape descriptors in Perez-Perez and this study. (DOCX) [file pone.0096889.s012.docx]

Table S4.Correspondence of nearest shape descriptors in Perez-Perez and this study.

| Perez-Perez, *et al*. Abbreviation | Descriptor Name and Comments | Camargo *et al*.  Name and comments |
| --- | --- | --- |
| RA | Rosette Area | Area |
| RP | Rosette Perimeter | Circumference |
| RXF | Maximum Feret’s diameter of rosette | Maxdiam |
| EA | Area of ellipse fitted to outline of rosette | Convexhullarea  Area of convex hull |
| EP | Perimeter of ellipse fitted to outline of rosette | Convexhullcirc  Perimeter of convex hull |
| RC | Rosette Compactness  Calculated as RA /EA | Compactness  Calculated as Area/ Convexhullarea |
| RE | Rosette Evenness  Calculated as RP/EP | Roundness  Calculated as Circumference^2^ / Area |
