# Supplementary material for: Objective Definition of Rosette Shape Variation Using a Combined Computer Vision and Data Mining Approach
Source: PLoS One. 2014 May 7;9(5):e96889. doi: 10.1371/journal.pone.0096889 (PMC4013065; doi:10.1371/journal.pone.0096889)
Supplement: Table S5 — Correlation coefficient between each descriptor and principal component one. (DOCX) [file pone.0096889.s013.docx]

Table S5. Correlation coefficient between each descriptor and dimension one. Correlation coefficient of the variables which are significantly correlated to the principal dimensions.

| Descriptor | Correlation Coefficient, r |
| --- | --- |
| RP | 0.989 |
| EP | 0.974 |
| RXF | 0.973 |
| EA | 0.962 |
| RA | 0.962 |
| RE | 0.799 |
| RC | 0.603 |
