# Supplementary material for: Objective Definition of Rosette Shape Variation Using a Combined Computer Vision and Data Mining Approach
Source: PLoS One. 2014 May 7;9(5):e96889. doi: 10.1371/journal.pone.0096889 (PMC4013065; doi:10.1371/journal.pone.0096889)
Supplement: Table S6 — Variable names used to store final results of calculations in the Matlab program on a binary image stored as bw. Also scale factor (sf) applied to convert units from pixel based values to measurements (where value is not a ratio then no entry). (DOCX) [file pone.0096889.s014.docx]

Table S6 Variable names used to store final results of calculations in the Matlab program on a binary image stored as bw. Also scale factor (sf) applied to convert units from pixel based values to measurements (where value is not a ratio then no entry).

This conversion allows

where scaled value =value in pixel units/(pixels per mm^sf^)

| Id | Parameter Name | Variable name in program | Scale factor sf | Units |
| --- | --- | --- | --- | --- |
| 1 | Mincirclediam | dc | 1 | mm |
| 2 | Normsmallpax | xmpslan |  | Ratio |
| 3 | Normlargepax | xpmalan |  | Ratio |
| 4 | Minrectarea | bbarea | 2 | mm^2^ |
| 5 | Mindistcenddy | comtb | 1 | mm |
| 6 | Vrectsizey | exty | 1 | mm |
| 7 | Vrectsizex | extx | 1 | mm |
| 8 | Compactness | comp |  | Ratio |
| 9 | Normrotmo | rotn |  | Ratio |
| 10 | Area | area | 2 | mm^2^ |
| 11 | Paxratio | Ratmaj2min3 |  | Ratio |
| 12 | Circumference | peri | 1 | mm |
| 13 | Excentricity | mectsq |  | Ratio |
| 14 | Maxdiam | res | 1 | mm |
| 15 | Roundness | rond |  | Ratio |
| 16 | Bdryround | bpr |  | Ratio |
| 17 | Bdrycount | bpc | 1 | mm |
| 18 | Bdrytoarearatio | bpa | -1 | mm^-1^ |
| 19 | Conhullcirc | lengg | 1 | mm |
| 20 | Mincirclediam | convarea | 2 | mm^2^ |
