# Supplementary material for: Objective Definition of Rosette Shape Variation Using a Combined Computer Vision and Data Mining Approach
Source: PLoS One. 2014 May 7;9(5):e96889. doi: 10.1371/journal.pone.0096889 (PMC4013065; doi:10.1371/journal.pone.0096889)
Supplement: Methods S1 — Description of calculations of shape parameters. (DOCX) [file pone.0096889.s015.docx]

# S1. Calculations of shape parameters.

The calculations described below are based on the images of the rosettes being segmented as white regions containing the rosette against a black background. Because the exact methods required to segment images will depend on the specific illumination, background and plant characteristics, this will not be described in detail here. These will need to be established in each laboratory aiming to use this approach and the specific method we have used is described in the paper.

The calculations were all performed on a per-pixel basis, and results calculated below were then converted to appropriately dimensioned values using pixel to mm conversion factors determined for each image processed using the scaling powers shown in Table 1. The aim of this was to simplify comparison between parameters calculated from images collected at different resolutions by various cameras, and the system used here had an average resolution of 7.3 pixels mm^-1^. Because of numerical effects in the calculations used the values derived will change slightly at much higher or lower resolutions.

A Matlab® program to perform the calculations below is available from the authors on request together with an example (TIF format image and results). The calculations require the Matlab® Image Processing Toolbox and the routines minboundcircle, minBoundingBox and optionally ellipseaxes downloaded from the Matlab® file exchange. These Matlab routines replicate the results from the LemnaTec software well with an average deviation of 0.006% and maximum deviation of 0.07% on the example image.

Parameters which are ratios of other parameters defined in Table 1 in the paper are not further described below.

**1.1 Parameters derived directly from Matlab regionprops command:**

Area, centroidx, centroidy, vrectsizex and vrectsizey were determined with the 'Area'**,**'Centroid'**,**'BoundingBox’ options on the image and selecting the appropriate outputs.

Circumference was calculated by subtracting 1 from the option **'Perimeter'** (to allow for an overlapped pixel)

**1.2 Intermediate values used in further calculations extracted using Matlab reginonprops command:**

'PixelList'**,** was used to extract the coordinates of all of the pixels in the rosette for “Moment” based calculations, see below

'MajorAxisLength'**,**'MinorAxisLength' were used to extract paxratio (the ratio of the lengths of the principal axes) using:

| $paxratio=\left( \frac{MajorAxisLength}{MinorAxisLength} \right)^{2}$ | 1 |
| --- | --- |

The larger (largepax) and smaller (smallpax) principle axis moment were calculated as:

| ${largepax=\frac{MajorAxis Length}{16}}^{2}.Area$ | 2 |
| --- | --- |
| $smallpax=\frac{{MajorAxis Length}^{2}}{16}.Area$ | 3 |

with their normalised versions (normlargepax and normsmallpax) calculated by dividing by Area^2^.

**1.3 Moment Calculations**

These are defined by the general equation:

| $m_{pq =}\frac{1}{Area}\sum_{1,n} \left( u \right)^{p}.\left( v \right)^{q}$ | 4 |
| --- | --- |

where *u* and *v* are the *x* and *y* coordinates of the rosette pixels after subtraction of their respective centroid coordinates . 1,n is the summation over all the pixels in the image as extracted by 'PixelList'**.** p and q are the indexes of the powers of u and v used to define the various values of m.

Using the above equation *m_11_, m_02_* and *m_20_* were calculated then:

| $excentricity=\left( \frac{\sqrt{\left( m_{20}{-m}_{02} \right){}^{2}{}{+4m}_{11}^{2}}}{m_{20}{+m}_{02}} \right)^{2}$ | 5 |
| --- | --- |

Then using a similar summation to the moment calculations

| $rotmo=\sum_{1,n} {u^{2}+v}^{2}$ | 6 |
| --- | --- |

and the normalised version:

| $normrotmo=\frac{rotmo}{{area}^{2}}$ | 7 |
| --- | --- |

**1.4 Calculations using morphological operators**

Morphological operators can perform many functions, see eg Gonzalez et al for a fuller description, here a morphological operator was used to either to reduce or enlarge the boundary of the rosette by one pixel. The morphological operator used was a 3 row by 3 column matrix of ones.

To calculate bdrycount the operator was used once on the original image of the rosette to reduce its boundary (erosion) by one pixel and the area of this figure was subtracted from that of the original figure, giving the pixel count of the rosette border. A combined image showing the boundary pixels was then created by subtraction, and the list of boundary pixels generated from it was used to calculate mindistcentbdy.

To calculate the mincirclediam the morphological operator was used to enlarge the rosette by one pixel (dilation) and the convex hull of this figure was calculated using the 'ConvexHull' option of the regionprops command and used as input to the minboundcircle function from the Matlab file exchange. This provided the radius, and hence the diameter of the circle that would just allow the object to pass through

**1.5 Calculations based on the Matlab® bwboundaries command**

The pixels forming the outer boundary of the rosette were extracted using the bwboundaries command, and the maxdiam was calculated as the maximum distance between these pixels. The boundary pixels were then used as input to the convhull command, which provided the area (conhullarea) and the list of pixels defining the convex hull. conhullcirc was then calculated by summing the distances between the pixels defining the convex hull. The coordinates of smallest rectangle of any orientation in which the rosette would fit was calculated from the outer boundary of the rosette using the minBoundingBox routine and the area of the rectangle (minrectarea) was calculated from these coordinates.
