# Supplementary material for: Objective Definition of Rosette Shape Variation Using a Combined Computer Vision and Data Mining Approach
Source: PLoS One. 2014 May 7;9(5):e96889. doi: 10.1371/journal.pone.0096889 (PMC4013065; doi:10.1371/journal.pone.0096889)
Supplement: Methods S2 — Compressed file contains a number of files. 1) Raw data. Descriptors extracted from each segmented image, 2) R scripts used to handle and analyse data, 3) Matlab scripts used as another way to extract the 20 shape descriptors used in this analysis, 4) Example image suitable for processing by the Matlab script and 5) File descriptions, summary of files in this zip file. (ZIP) [file pone.0096889.s016.zip › filedescription.docx]

**File description**

| Name | Type | Description |
| --- | --- | --- |
| plosone_rawdata | csv | Raw data |
| plosone_arabmagic | R script | Script to analyse raw data |
| plosone_temp | R script | Script to analyse raw data |
| jsupp2 | Matlab Script | Contain equations to calculate descriptors. |
| t1_e1_bw | tiff | Contain image to test jsupp2 script |
